# Supplementary material for: Structure of the Helicase Domain of DNA Polymerase Theta Reveals a Possible Role in the Microhomology-Mediated End-Joining Pathway
Source: Structure. 2015 Dec 1;23(12):2319–30. doi: 10.1016/j.str.2015.10.014 (PMC4671958; doi:10.1016/j.str.2015.10.014)
Supplement: Document S1. Supplemental Experimental Procedures, Figures S1–S4, and Table S1 [file mmc1.pdf]

Structure, Volume *23*

Supplemental Information

Structure of the Helicase Domain of DNA Polymerase Theta Reveals a Possible Role in the Microhomology-Mediated End-Joining Pathway

Joseph A. Newman, Christopher D.O. Cooper, Hazel Aitkenhead, and Opher Gileadi

Supplementary Material

Supplementary Figures

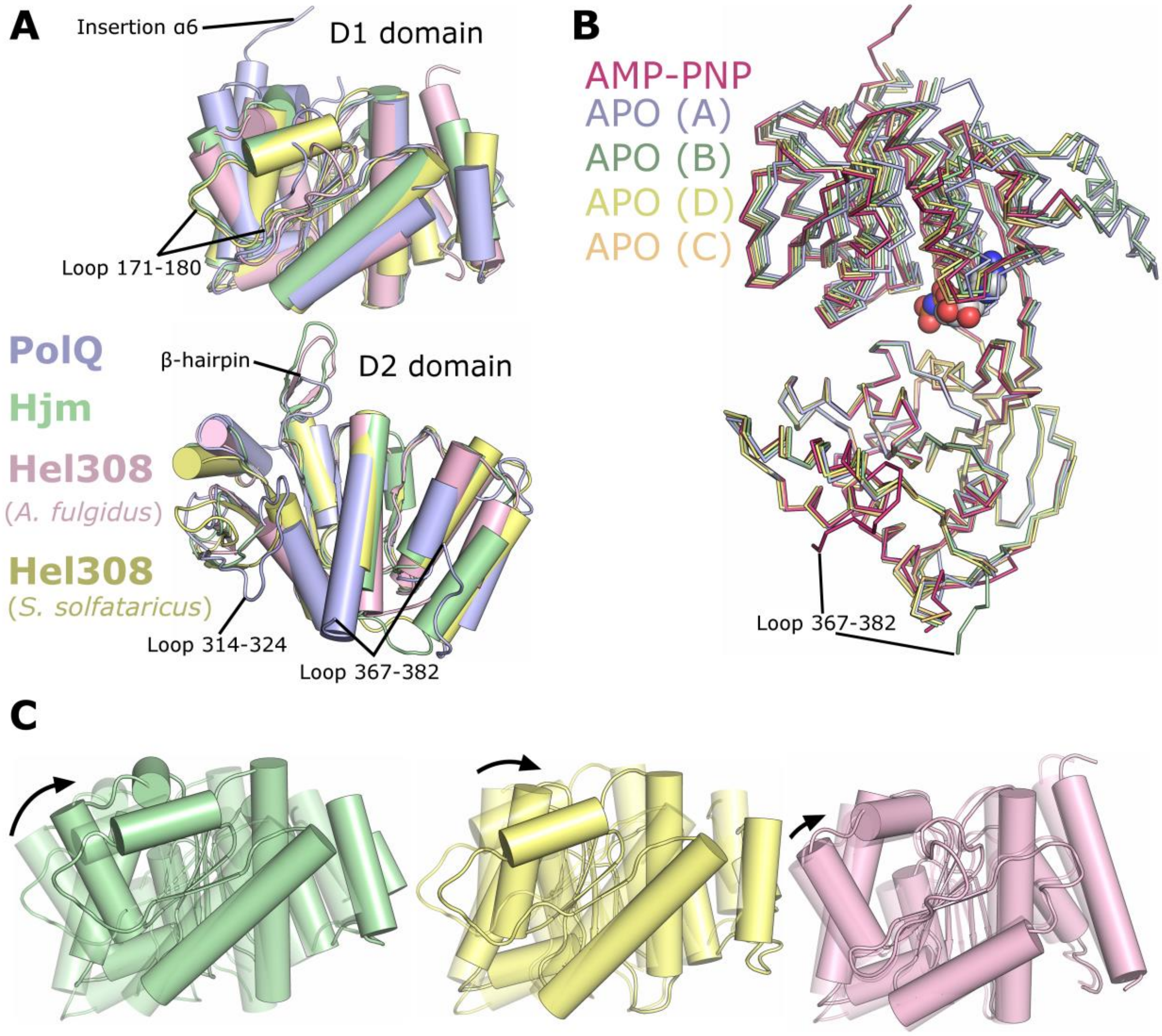

Figure S1, related to Figure 1- Comparison of Polθ-HLD core with archaeal Hel308/Hjm proteins. (**A**) Structural superposition of Polθ-HLD (blue), Hjm (green), *A. fulgidus* Hel308 (pink), and *S. solfataricus* Hel308 (yellow), on the basis of the D1 (upper) and D2 (lower) domains, with variable regions labelled. (**B**) Comparison of the D1-D2 conformation of the nucleotide and the 4 chains of the APO protein. All structures were superposed on the basis of the D2 domain alone. (**C**) Comparison of the D1-D2 domain conformations of Hjm (green), *S. solfataricus* Hel308 (yellow) and *A. fulgidus* Hel308 (pink) with PolQ. The semi-transparent image shows the position of the D1 domain when a structural superposition to PolQ is performed on the basis of the D2 domain alone, the solid image shows the altered position when superposed on the basis of the D1 domain alone. The black arrow shows an approximate vector describing the relative domain motion (shorter arrow is more similar conformation).

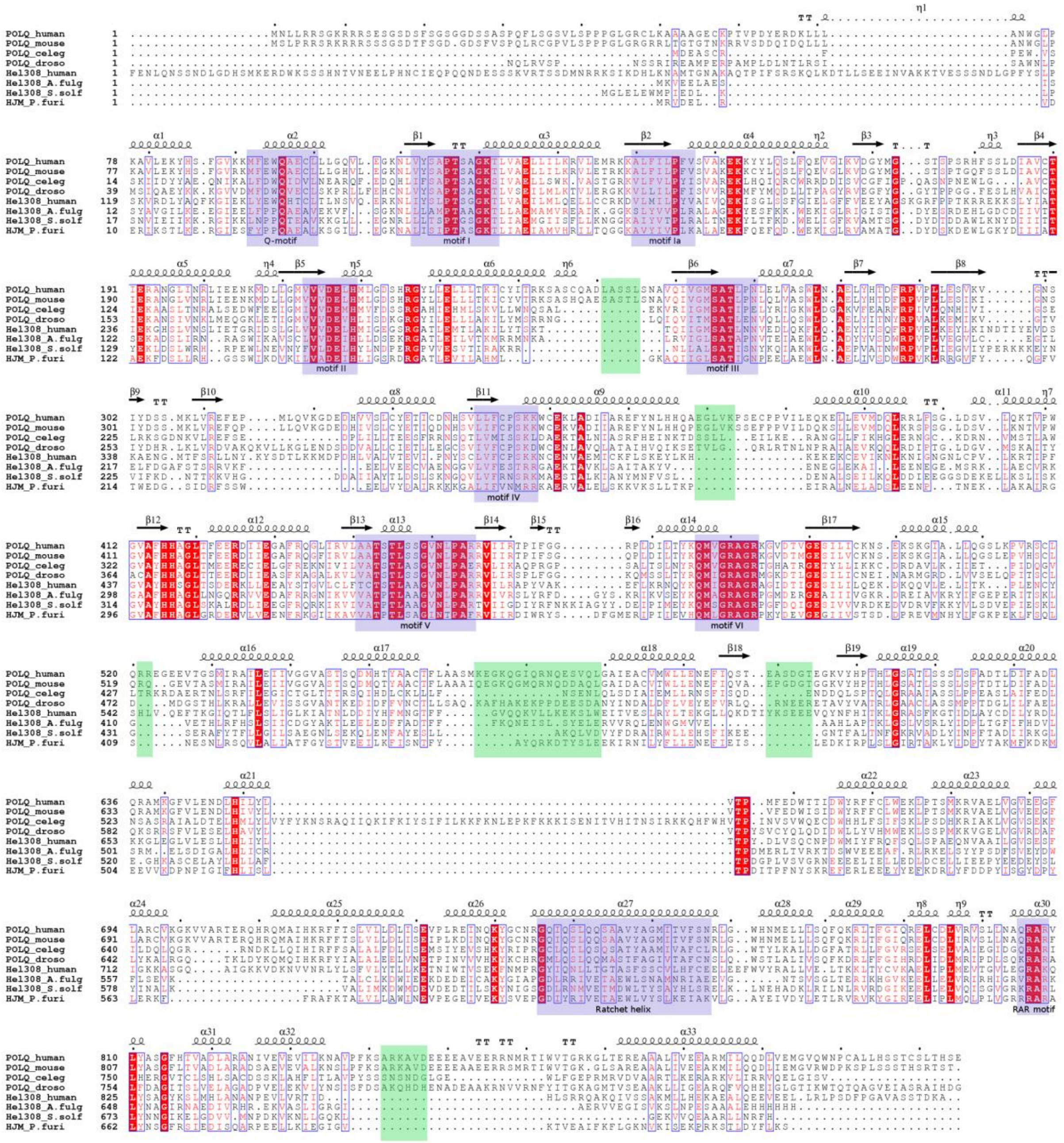

Figure S2, related to Figure 1B – Multiple sequence alignment of Polθ-HLD and selected homologues. Multiple sequence alignment was performed using CLUSTALW, and displayed using ESCRIPT. Secondary structural elements from the Polθ-HLD AMP-PNP structure (PDBid 5AGA) are displayed on top with important helicase regions highlighted in purple, and regions disordered in the 5AGA structure highlighted in green.

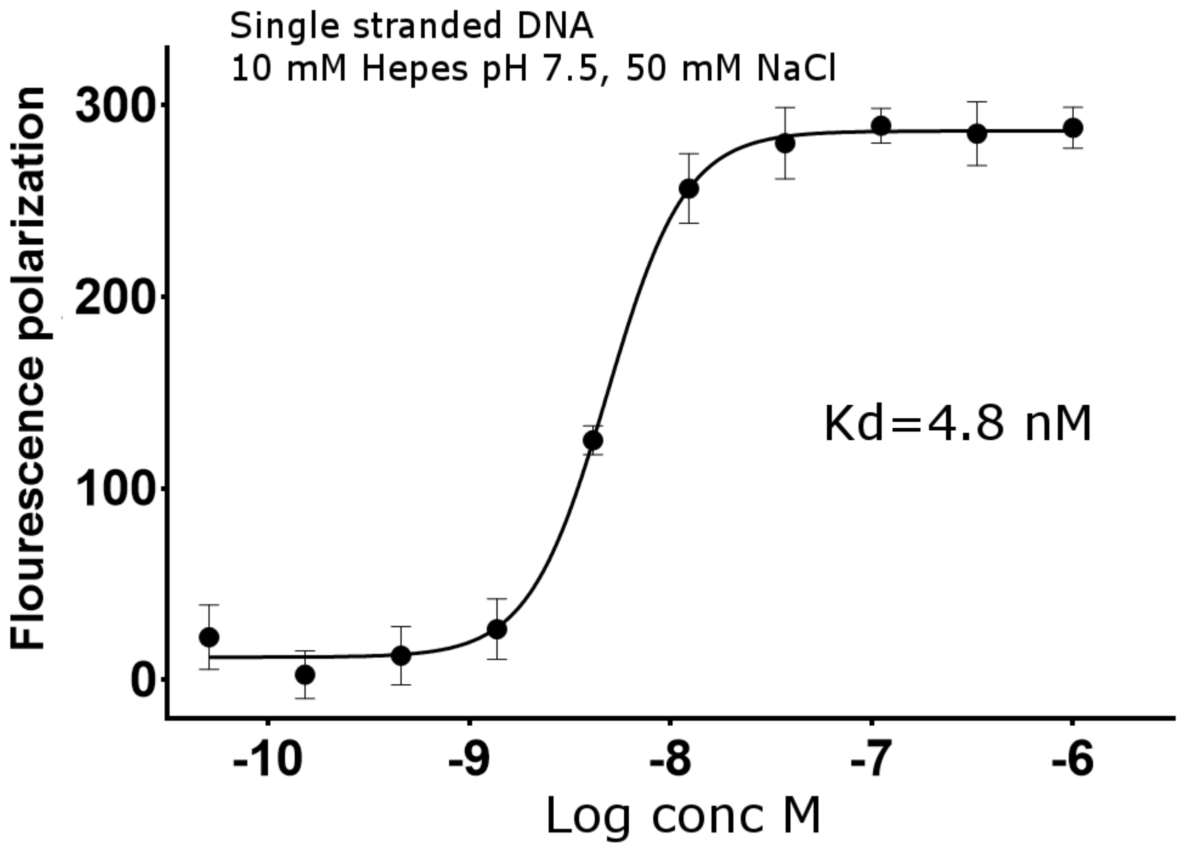

Figure S3, related to Figure 5- Polθ-HLD binding to single stranded DNA in low salt buffer. Error bars are plotted ± standard error of at least three independent replicates.

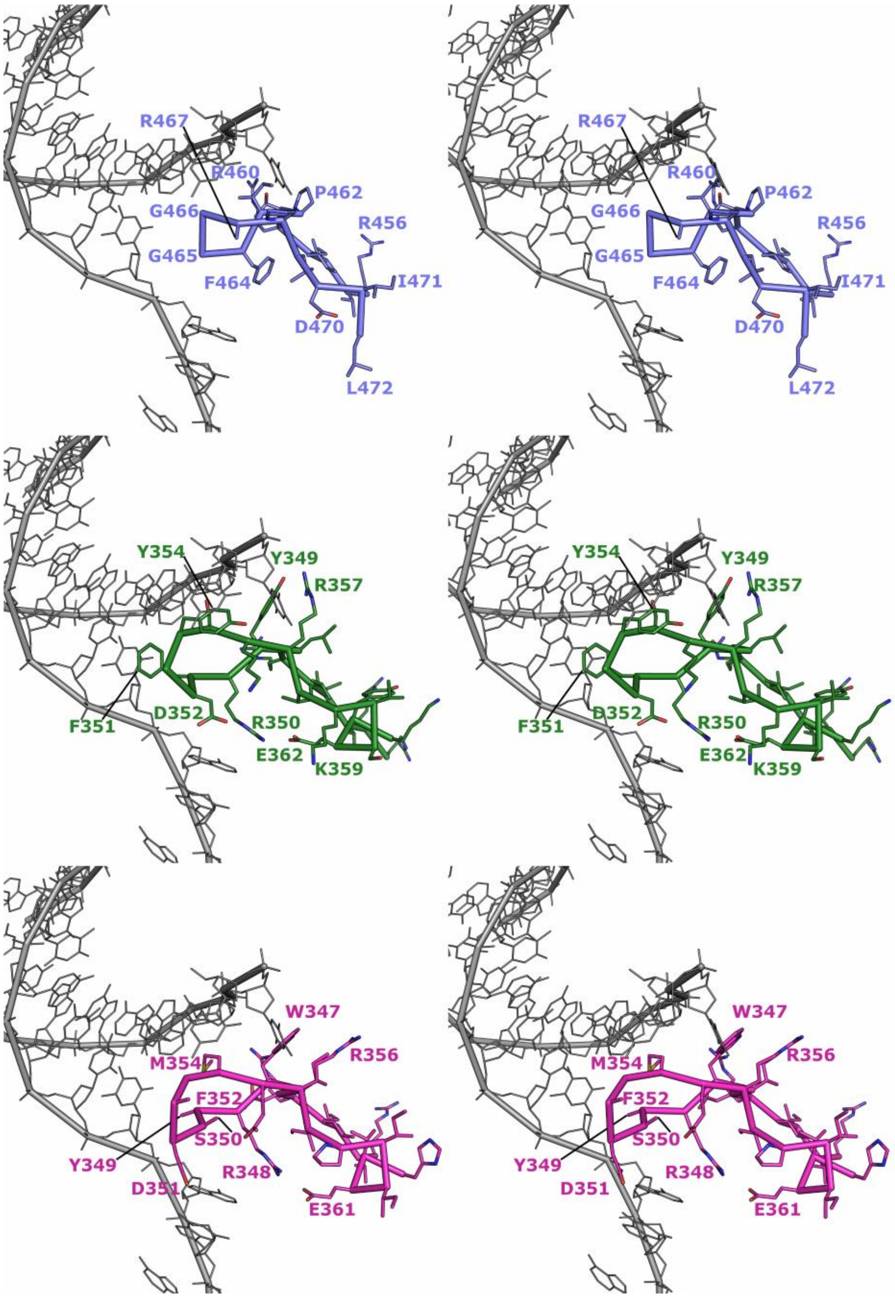

Figure S4, related to Figure 6- Comparison of the β-hairpins of Polθ-HLD, *A. fulgidus* Hel308 and Hjm. Stereo view of the β-hairpin regions of Polθ-HLD (blue, upper panel), *A. fulgidus* Hel308 (green, centre panel) and Hjm (pink, lower panel) with key residues labelled. The DNA from the *A. fulgidus* Hel308 DNA complex is shown in grey for reference throughout.

Table S1A, related to Experimental Procedures: DNA-binding assays. Sequences of oligonucleotides used in the fluorescence polarisation DNA binding assay

Oligonucleotide name	Sequence
POLQ-1	*5'ATCGATAGTCGGATCCTCTAGACAGCTCCATGTAGCAAGGCACTGGTAGAATTCGGCAGCGTC (63 bases)
POLQ-2	5'GACGCTGCCGAATTCTACCAGTGCCTTGCTACATGGAGCTGTCTAGAGGATCCGACTATCGAT (63 bases)
POLQ-3	5'GACGCTGCCGAATTCTACCAGTGCCTTGCTAGGACATCTTTGCCCACCTGCAGGTTCACCC (61 bases)
POLQ-4	5'GGAGCTGTCTAGAGGATCCGACTATCGA (28 bases)
POLQ-5	5'TGGGTGAACCTGCAGGTGGGCAAAGATG (28 bases)
POLQ-6	5'GGGTGAACCTGCAGGTGGGCAAAGATGTCCATTAGTGGATCCTTAGCACCGTTGTAAGACG (61 bases)
POLQ-7	5'CGTCTTACAACGGTGCTAAGGATCCACTAATCATGGAGCTGTCTAGAGGATCCGACTATCGAT (63 bases)
POLQ-8	5'GACGCTGCCGAATTCTACCAGTGCCTTGCTATTTTTTTTTTTTTCATGGAGCTGTCTAGAGGATCCGACTATCGAT (75 bases)
POLQ-9	5'GACGCTGCCGAATTCTACCAGTGCCTTG (28 bases)

* Oligonucleotide POLQ-1 was obtained with a Fluorescein isothiocyanate label on the 5' end

Table S1B, related to Experimental Procedures: DNA-binding assays. Mixing scheme for DNA substrates

Substrate name	Structure	Oligonucleotides
Single stranded	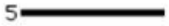	POLQ-1
Double stranded	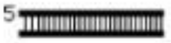	POLQ-1 + POLQ-2
3′ Overhang	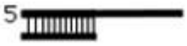	POLQ-1 + POLQ-4
Splayed duplex	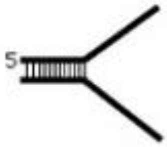	POLQ-1 + POLQ-3
3′ Flap	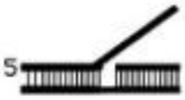	POLQ-1 + POLQ-3 + POLQ-4
3 way junction	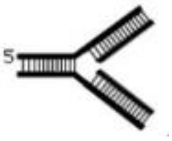	POLQ-1 + POLQ-3 + POLQ-4 + POLQ-5
4 Way junction	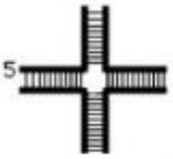	POLQ-1 + POLQ-3 + POLQ-6 + POLQ-7
Looped duplex	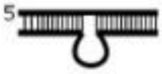	POLQ-1 + POLQ-8
5′ overhang	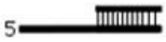	POLQ-1 + POLQ-9

In all cases the fluorophore was attached to the 5′ end of oligo POLQ-1 in the position marked by the 5 on the structure diagrams.

Supplemental Experimental Procedures

Protein expression and purification.

The Polθ-HLD constructs (residues 1-894 and 67-894) were cloned in to the baculovirus transfer vector pFB-LIC-Bse and transformed into *E. coli* DH10Bac to generate recombinant bacmids. Spodoptera frugiperda (Sf9) cells were infected with P2 virus (2x 10^6 cells/ml), and proteins were expressed in 1 litre cultures at 27°C for 72hrs and harvested by centrifugation. For purification cell pellets were thawed and resuspended in buffer A (50 mM HEPES pH 7.5, 500 mM NaCl, 5% glycerol, 10 mM imidazole, 0.5 mM Tris (2-carboxyethyl) phosphene (TCEP)), with the addition of 1x protease inhibitor set VII (Merck, Darmstadt, Germany). Cells were lysed using sonication and cell debris pelleted by centrifugation. Lysates were applied to a Ni-IDA IMAC gravity flow column, washed with 2 column volumes of wash buffer (buffer A supplemented with 30 mM imidazole), and eluted with the addition of 300 mM imidazole in buffer A. The purification tag was cleaved with the addition of 1:20 mass ratio of His-tagged TEV protease during overnight dialysis into buffer B (20 mM HEPES, pH 7.5, 500 mM NaCl, 5 % glycerol, 0.5 mM TCEP). TEV was removed by IMAC column rebinding and final protein purification was performed by size exclusion chromatography using a HiLoad 16/60 Superdex 200 column at 1 ml/min in buffer B. Protein concentrations were determined by measurement at 280nm (Nanodrop) using the calculated molecular mass and extinction coefficients.

Crystallization and Structure Determination

For crystallization Polθ-HLD was concentrated to 10 mg/ml using a 50,000 mwco centrifugal concentrator and exchanged to a buffer containing 10 mM HEPES pH 7.5, 250 mM NaCl, 0.5 mM TCEP. AMP-PNP and ADP bound crystals were obtained at 20°C from conditions containing 19% PEG 3350, 0.2M potassium citrate tribasic pH 8.5, 10 mM $MgCl_2$ and 10 mM of either AMP-PNP or ADP respectively. The *apo* protein was concentrated to 15 mg/ml and crystallized at 20°C from conditions containing 0.2 M NaCl, 0.1 M HEPES pH 7.0, 20 % PEG 6K and 10% ethylene glycol. Crystals were cryo-protected by transferring to a solution of mother liquor supplemented with 25 % ethelene glycol and flash-cooled in liquid nitrogen. Data were collected at diamond light source beamline I04-1 (AMP-PNP and ADP) and I03 (*apo*). Diffraction data were processed with the program XDS(Kabsch, 2010), and the structures were solved by molecular replacement using the program PHASER(McCoy et al., 2007) with *P.furiosis* Hjm(Oyama et al., 2009) structure as a starting model. Model building and real space refinement were performed in COOT(Emsley et al., 2010) and the structures refined using PHENIX REFINE(Adams et al., 2010). The structural coordinates and structure factors were

deposited in the Protein Data Bank, PDB: 5A9J (apo), 5AGA (AMP-PNP) and 5A9F (ADP). Structure validation summary reports can be retrieved from the PDB entries.

Analytical Size Exclusion Chromatography

Analytical size exclusion chromatography with on-line multiangle light scattering (SEC-MALS) was performed on a Viscotek TDA 305 system (Malvern Instruments Ltd) using a 15-ml Shodex KW-803 silica column (GE healthcare). 50 µl of the respective proteins were injected on to the column which was run at a flow rate of 0.5 ml/min in a buffer containing 20 mM HEPES 7.5, 5% glycerol, 500 mM NaCl and 0.5 mM TCEP. Calibration was performed using the molecular weight standards thyroglobulin (670,000 Da), ɣ-globulin (158,000 Da), ovalbumin (44,000 Da), myoglobin (17.000 Da) and vitamin B_{12} (1,350 Da), using the same buffer and flow rate. MALS data were analysed using the Omnisec software (Malvern Instruments Ltd) using Bovine serum albumin as an internal calibration sample.

Analytical Ultracentrifugation

Sedimentation velocity AUC experiments were performed on a XL-I Analytical Ultracentrifuge using a Ti-50 rotor and cells with double-sector centrepieces (Beckman Coulter, Brea, CA, USA). Experiments were performed with 0.35 mg/ml protein in 20 mM HEPES (pH 7.5), 150 mM NaCl, and 0.5 mM TCEP. Samples were filtered through a 0.22 µm syringe filter prior to analysis, employing a rotor speed of 40,000 rpm at 20°C, against a matched buffer reference. Absorbance (280 nm) and interference data were analyzed with SEDFIT(Schuck, 2000) to calculating c(s) distributions, and using partial specific volumes and buffer parameters calculated using the program SEDNTERP(Harding et al., 1992). Theoretical sedimentation coefficients of model proteins were calculated from Polθ-HLD structures using the program HYDROPRO(Ortega et al., 2011).

Small angle X-ray scattering

Small angle X-ray scattering measurements of Polθ-HLD in solution were performed at Diamond light source beamline B21 using a BIOSAXS robot for sample loading. Measurements were made using protein concentrations of 2.5, and 1.25 mg/ml in a buffer comprising 20 mM HEPES pH 7.5, 250 mM NaCl, 0.5 mM TCEP, 1 mM ADP. The data were reduced and buffer contributions subtracted with the DawnDiamond software suite and analysed using the program SCATTER (www.bioisis.net). Real space scattering profiles of atomic models were calculated from atomic models using CRYSOL(Svergun et al., 1995) and aligned and scaled to the experimental data using PRIMUS(Konarev et al., 2003). The agreement between theoretical and experimental scattering profiles was evaluated using the χ^2 free procedure(Rambo and Tainer, 2013) implemented in the program SCATTER.

DNA binding assays

DNA binding was measured using a fluorescence polarisation based assay. DNA oligonucleotide substrates were prepared by mixing the oligonucleotide sequences and combinations listed in table S1. For all substrates the master strand was labelled on the 5' end with Fluorescein isothiocyanate and oligonucleotides were mixed in the ratio 1:1.1 (slight excess on unlabelled oligonucleotide) at 5 mM final concentration in a buffer consisting of 10 mM HEPES pH 7.5, 50 mM NaCl before heating to 96°C and allowing to cool on a heat block over 2 hrs. Probes were used at a final concentration of 10 nM and binding experiments were performed in a buffer containing 10 mM HEPES pH 7.5, 150 mM NaCl. Measurements were performed in 96 well plates (200 µl volume) in a POLARstar omega plate reader (BMG Labtech). Kinetic constants were calculated from binding curves using a 4 parameter logarithmic binding equation using the program PRISM (GraphPad).

ATPase activity assays

ATPase activity of Polθ-HLD was measured using a pyruvate kinase, lactate dehydrogenase enzyme linked absorbance assay(Norby, 1988) in which the loss of absorbance at 340 nM (6,250 cm^{-1} M^{-1}) is coupled to ATP hydrolysis. The reaction mixtures (40 ul) contained 0.85 U Lactate Dehydrogenase, 4.2 U Pyruvate Kinase, 0.2 mM NADH, 0.5 mM Phosphoenolpyruvate, 20 mM HEPES pH 7.5, 200 mM NaCl, and ATP concentrations between 3 µM and 0.8 mM. For DNA stimulated ATPase the reaction mix contained in addition 2 µM of single stranded 18 bp DNA (ACTCGTCTCTAGCTTTTT). Reactions were initiated by the addition of protein (0.1 µM with DNA and 3.4 µM without) and measured over 15 minutes in a POLARstar omega plate reader (BMG Labtech). Kinetic constants were calculated from binding curves using a 4 parameter logarithmic binding equation using the program PRISM (GraphPad).

References

Adams, P.D., Afonine, P.V., Bunkoczi, G., Chen, V.B., Davis, I.W., Echols, N., Headd, J.J., Hung, L.W., Kapral, G.J., Grosse-Kunstleve, R.W.*, et al.* (2010). PHENIX: a comprehensive Python-based system for macromolecular structure solution. Acta Crystallogr D Biol Crystallogr *66*, 213-221.
Emsley, P., Lohkamp, B., Scott, W.G., and Cowtan, K. (2010). Features and development of Coot. Acta crystallographica Section D, Biological crystallography *66*, 486-501.
Harding, S.E., Rowe, A.J., and Horton, J.C. (1992). Analytical ultracentrifugation in biochemistry and polymer science ([Cambridge]: Royal society of chemistry).
Kabsch, W. (2010). Xds. Acta crystallographica Section D, Biological crystallography *66*, 125-132.
Konarev, P.V., Volkov, V.V., Sokolova, A.V., Koch, M.H.J., and Svergun, D.I. (2003). PRIMUS: a Windows PC-based system for small-angle scattering data analysis. Journal of applied crystallography *36*, 1277-1282.
McCoy, A.J., Grosse-Kunstleve, R.W., Adams, P.D., Winn, M.D., Storoni, L.C., and Read, R.J. (2007). Phaser crystallographic software. Journal of applied crystallography *40*, 658-674.
Norby, J.G. (1988). Coupled Assay of Na+,K+-Atpase Activity. Method Enzymol *156*, 116-119.

Ortega, A., Amoros, D., and Garcia de la Torre, J. (2011). Prediction of hydrodynamic and other solution properties of rigid proteins from atomic- and residue-level models. Biophysical journal *101*, 892-898.
Oyama, T., Oka, H., Mayanagi, K., Shirai, T., Matoba, K., Fujikane, R., Ishino, Y., and Morikawa, K. (2009). Atomic structures and functional implications of the archaeal RecQ-like helicase Hjm. BMC structural biology *9*, 2.
Rambo, R.P., and Tainer, J.A. (2013). Accurate assessment of mass, models and resolution by small-angle scattering. Nature *496*, 477-481.
Schuck, P. (2000). Size-distribution analysis of macromolecules by sedimentation velocity ultracentrifugation and lamm equation modeling. Biophysical journal *78*, 1606-1619.
Svergun, D., Barberato, C., and Koch, M.H.J. (1995). CRYSOL - A program to evaluate x-ray solution scattering of biological macromolecules from atomic coordinates. Journal of applied crystallography *28*, 768-773.
