# Supplementary material for: Structure of the Helicase Domain of DNA Polymerase Theta Reveals a Possible Role in the Microhomology-Mediated End-Joining Pathway
Source: Structure. 2015 Dec 1;23(12):2319–30. doi: 10.1016/j.str.2015.10.014 (PMC4671958; doi:10.1016/j.str.2015.10.014)
Supplement: Document S2. Article plus Supplemental Information [file mmc2.pdf]

Structure

Article

Structure of the Helicase Domain of DNA Polymerase Theta Reveals a Possible Role in the Microhomology-Mediated End-Joining Pathway

Graphical Abstract

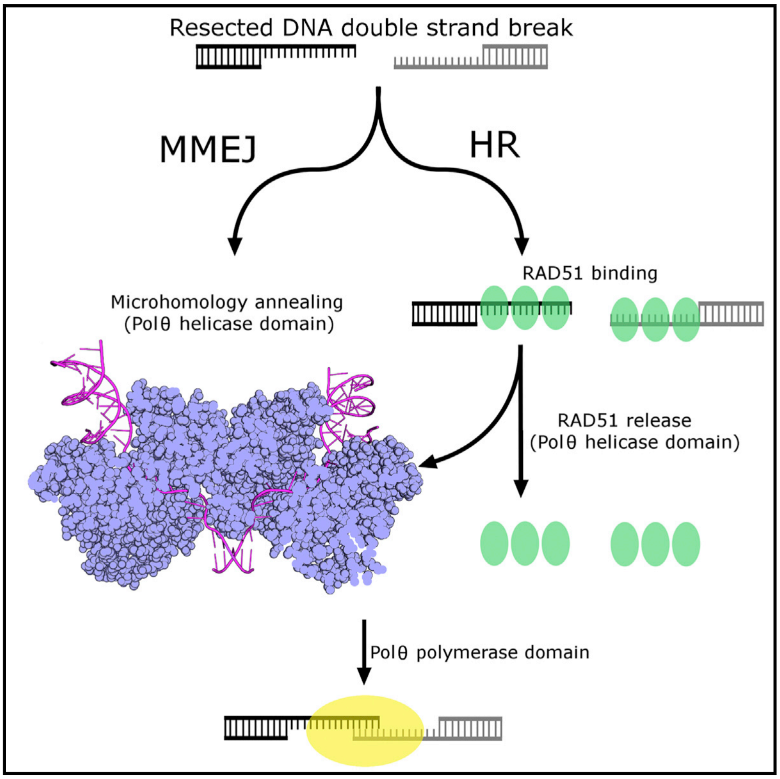

Highlights

- DNA polymerase theta (Polθ) is unique in having a DNA helicase-like domain
- Polθ has a role in the repair of DNA double-strand breaks and in cancer cell survival
- We solved the structure of the tetrameric helicase-like domain
- The tetramer may align broken DNA ends for microhomology-mediated end joining

Authors

Joseph A. Newman, Christopher D.O. Cooper, Hazel Aitkenhead, Opher Gileadi

Correspondence

opher.gileadi@sgc.ox.ac.uk

In Brief

We describe a structure of an unusual helicase domain of DNA polymerase theta (Polθ). The helicase-like domain of Polθ forms tetramers and this tetrameric structure might bring together broken DNA ends for microhomology-mediated end joining.

Newman et al., 2015, Structure *23*, 2319–2330
December 1, 2015 ©2015 The Authors
http://dx.doi.org/10.1016/j.str.2015.10.014

CellPress

Structure of the Helicase Domain of DNA Polymerase Theta Reveals a Possible Role in the Microhomology-Mediated End-Joining Pathway

Joseph A. Newman,[1,2] Christopher D.O. Cooper,[1,2,3] Hazel Aitkenhead,[1] and Opher Gileadi[1,*]
[1]Structural Genomics Consortium, University of Oxford, ORCRB, Roosevelt Drive, Oxford OX3 7DQ, UK
[2]Co-first author
[3]Present address: Department of Biological Sciences, School of Applied Sciences, University of Huddersfield, Huddersfield, West Yorkshire HD1 3DH, UK
*Correspondence: opher.gileadi@sgc.ox.ac.uk
http://dx.doi.org/10.1016/j.str.2015.10.014
This is an open access article under the CC BY license (http://creativecommons.org/licenses/by/4.0/).

SUMMARY

DNA polymerase theta (Polθ) has been identified as a crucial alternative non-homologous end-joining factor in mammalian cells. Polθ is upregulated in a range of cancer cell types defective in homologous recombination, and knockdown has been shown to inhibit cell survival in a subset of these, making it an attractive target for cancer treatment. We present crystal structures of the helicase domain of human Polθ in the presence and absence of bound nucleotides, and a characterization of its DNA-binding and DNA-stimulated ATPase activities. Comparisons with related helicases from the Hel308 family identify several unique features. Polθ exists as a tetramer both in the crystals and in solution. We propose a model for DNA binding to the Polθ helicase domain in the context of the Polθ tetramer, which suggests a role for the helicase domain in strand annealing of DNA templates for subsequent processing by the polymerase domain.

INTRODUCTION

DNA polymerase theta (Polθ, encoded by the *POLQ* gene) is one of ∼15 DNA polymerases in the human genome, first identified by homology to the *Drosophila mus308* gene, with *mus308* mutants exhibiting hypersensitivity to DNA interstrand crosslinking agents (Sharief et al., 1999). Polθ is distinct among human DNA polymerases, exhibiting not only a C-terminal DNA polymerase domain (Seki et al., 2003; Shima et al., 2003; Yousefzadeh and Wood, 2013) but also an N-terminal domain exhibiting similarity and motifs typical of superfamily 2 (SF2) helicases (Singleton et al., 2007), separated by a long and lesser-conserved central domain of unknown function (Seki et al., 2003; Shima et al., 2003; Yousefzadeh and Wood, 2013) (Singleton et al., 2007). *POLQ* genes are widespread in multicellular eukaryotes (Yousefzadeh and Wood, 2013), including higher plants but not in fungi (Inagaki et al., 2006). Polθ is one of three human Family A DNA polymerases (Braithwaite and Ito, 1993) but, unusually within this family, it exhibits a very low fidelity of synthesis on normal DNA (Arana et al., 2008) and can bypass thymine glycol oxidative lesions (Seki et al., 2004; Yoon et al., 2014). Unusually, it can both insert opposite, and extend from, an abasic (AP) site (Seki et al., 2004), and the non-canonical DNA polymerase fold of Polθ influences processivity and translesion synthesis (Hogg et al., 2011; Zahn et al., 2015). Furthermore, the ability of Polθ to extend single-stranded DNA (ssDNA) (Hogg et al., 2012) and its 5′ deoxyribosephosphate lyase activity (Prasad et al., 2009) point to roles in various DNA repair pathways (Hogg et al., 2011, 2012; Prasad et al., 2009; Seki et al., 2004; Seki and Wood, 2008; Yoon et al., 2014).

Recent studies have established Polθ as playing a central role in an alternative DNA double-strand break repair process named the microhomology-mediated end-joining (MMEJ) pathway (Ceccaldi et al., 2015; Kent et al., 2015; Mateos-Gomez et al., 2015; Yousefzadeh et al., 2014). MMEJ is a mutagenic and error prone alternative to homologous recombination (HR) or non-homologous end-joining that utilizes short (2–6 bp) microhomologies to join the two strands (McVey and Lee, 2008), an activity that is unique to Polθ (Kent et al., 2015). MMEJ is generally not the preferred method of double-strand break repair in healthy cells, although it is increasingly important in cells deficient in HR (Cancer Genome Atlas Research Network, 2011). Polθ is overexpressed in certain cancers (Ceccaldi et al., 2015; Higgins et al., 2010a; Kawamura et al., 2004), correlating with poor survival in breast cancers (Higgins et al., 2010a; Lemee et al., 2010). Depletion of Polθ leads to hypersensitivity to radiation (Goff et al., 2009; Higgins et al., 2010b; Yousefzadeh et al., 2014), and decreased survival of cells deficient in homology-directed repair (Ceccaldi et al., 2015).

While the DNA polymerase activity of Polθ has been extensively studied, less is known about the functions of the helicase domain. The Polθ helicase domain (Polθ-HLD) is a member of the SF2 helicases, most closely related to the Ski2/Hel308 family of helicases, which are involved in the ATP-dependent 3′-5′ unwinding of lagging strands on replication forks (Tafel et al., 2011; Buttner et al., 2007; Guy and Bolt, 2005), thought to facilitate replication restart at stalled or damaged forks. Biochemical characterization of Polθ-HLD showed DNA-stimulated ATPase activity but failed to show any helicase activity (Ceccaldi et al., 2015; Maga et al., 2002; Seki et al., 2003). Thus, the role of the helicase domain in MMEJ is uncertain, as it was recently shown

that the polymerase domain alone is capable of performing the majority of the enzymatic steps of MMEJ in vitro (Kent et al., 2015). One clue as to the role of the helicase domain is the demonstration of the anti-recombinase activity of Polθ (Ceccaldi et al., 2015), with depletion of Polθ leading to an increase in RAD51 foci, which could be complemented by Polθ constructs lacking the polymerase domain. Polθ was found to interact directly with RAD51 (Ceccaldi et al., 2015), displacing RAD51 from ssDNA in a Polθ ATPase-dependent manner. The interaction was mapped to three regions in Polθ, two in the linker domain and one ~50 amino acid region in the C terminus of the helicase domain (Ceccaldi et al., 2015). Polθ depletion reduced survival of HR-deficient cells exposed to PARP, cisplatin, or MMC inhibitors, and this toxicity can be rescued by Polθ constructs lacking the polymerase domain but not by constructs lacking the RAD51 interacting motifs in the helicase domain (Ceccaldi et al., 2015). This points to Polθ-HLD as a potential target for cancer therapy to potentiate synthetic lethality in chemotherapeutic approaches (Shaheen et al., 2011).

In this study we present the crystal structure of *apo* and nucleotide-bound forms of Polθ-HLD. In common with the recently derived structure of the Polθ polymerase domain (Zahn et al., 2015), the structure displays numerous features based on insertions in the amino acid sequence that appear to be responsible for its unique activities. We find that Polθ-HLD exists as a tetramer in solution and consider the implications of this quaternary structure with regard to template preparation in the MMEJ pathway.

RESULTS

Overall Structure of the Polθ-HLD

We determined the structure of Polθ-HLD (residues 67–894), in a complex with the non-hydrolysable ATP analogue AMP-PNP to 2.9-Å resolution, and with ADP to 3.2-Å resolution, both in the same crystal form with a single copy of Polθ-HLD in the asymmetric unit. Using a slightly longer construct (residues 1–894), we obtained crystals of the *apo* protein, which diffracted to 3.55-Å resolution in a different crystal form containing four copies of Polθ-HLD in the asymmetric unit. Slight differences are seen in the ordering and positioning of various loops that are probably a result of local crystal contacts, as well as minor differences in domain orientation with the nucleotide-bound complexes (discussed later). A full summary of the model quality and refinement statistics are given in Table 1.

The overall structure of Polθ-HLD is most similar to the archaeal helicases exemplified by HEL308 from *Archaeoglobus fulgidus* and *Sulfolobus solfataricus* (Buttner et al., 2007; Richards et al., 2008) and Hjm from *Pyrococcus furiosus* (Oyama et al., 2009) (which share ~30% sequence identity with Polθ-HLD) and can be aligned with a root mean square deviation (RMSD) of around 2.6 Å over approximately 600 Cα residues (Figure S1A). Like its archaeal homologs, Polθ-HLD comprises five subdomains including the two core helicase domains and three additional, closely associated globular domains (Figure 1A). The N- and C-terminal RecA-like domains (D1 and D2), spanning residues 1–289 and 290–513, respectively, share a prototypical fold similar to *Escherichia coli* RecA, and contain the core machinery required for helicase activity, including ssDNA-binding motifs, the nucleotide-binding site, and all of the core helicase motifs (motifs I–VI) that are conserved across SF2 helicases (Figures 1B and S2). In the *apo* crystals, 32 additional residues can be seen at the N terminus. These residues form an extended loop and α-helix (only fully ordered in chain A, due to crystal packing), which packs against the rest of the D1 domain and is unique among the structures of SF2 helicases solved to date (Figures 1A and 1B). Other atypical features in the D1 domain include an unusual conformation of the loop connecting the third and fourth β strands (residues 171–180), which adopts a conformation similar to the equivalent loop in the Hel308 DNA complex (Buttner et al., 2007) (Figure S1A), and a 12-residue insertion at the C-terminal end of α6 (residues 238–260), which is somewhat disordered in both the *apo* and nucleotide-bound complexes (Figures 1B and S1A).

The D2 domain of Polθ-HLD also contains a number of inserted regions distinct from other helicase structures, most notably the loop between the third β strand and first α helix (residues 314–324), and the loop between the second and third helices on the D2 domain (residues 367–382) (Figures 1B and S1B). The conformation of the latter loop differs between the *apo* and nucleotide-bound crystals, and although sections of this loop are disordered in both crystals (and to a different extent in the various chains of the *apo* crystals), its conformation in the nucleotide-bound crystals may be a result of a citrate anion from the crystallization cocktail and is bound in the path of the loop, making polar contacts to nearby residues.

The Polθ-HLD Nucleotide-Binding Site

In the nucleotide-bound complexes, AMP-PNP and ADP bind in the nucleotide-binding site in a similar conformation to that observed in other helicase complexes. The electron density for the AMP-PNP molecule is significantly weaker than in the ADP complex (Figure 1C), especially around the adenine and ribose moieties, suggesting only partial occupancy in these crystals. The adenine moiety is hydrogen bonded to the conserved glutamate of the helicase motif 0 or "Q motif," although in Polθ-HLD the adenine moiety stacks against V89 and F93 rather than the conserved Leu and Arg residues common to the RecQ family. The α, β, and γ phosphates are in the equivalent positions as found in other helicase nucleotide complexes with conserved polar contacts formed to resides in the helicase motif I (Figure 1D). Similarly the Mg^{2+} ion is located in a similar position to that observed in other helicase nucleotide complexes (Bernstein et al., 2003; Pike et al., 2009), although in Polθ-HLD only a single contact with the γ phosphate is made, presumably due to the fact that the angle of the phosphorus nitrogen linkage places the oxygens of the β and γ phosphates slightly too distant to be bridged by a single Mg^{2+} ion.

Structural studies on other SF2 family helicases have established a model for the basic helicase mechanism whereby the binding, hydrolysis, and subsequent release of the nucleotide induces sequential conformational changes that lead to differences in the relative orientations of the ssDNA-binding regions of the D1 and D2 domains, which, when coupled to the molecular ratcheting of the ATP hydrolysis, lead to a single base translocation along ssDNA in the 3′-5′ direction (Gyimesi et al., 2010; Velankar et al., 1999). Comparing the relative orientations of the D1 and D2 domains in the *apo* and nucleotide-bound Polθ-HLD

Table 1. Data Collection and Refinement Statistics

	AMP-PNP Complex	ADP Complex	Polθ-HLD *apo*
Space group	I 2 2 2	I 2 2 2	P 1 2_1 1
Cell dimensions, *a*, *b*, *c* (Å)	116.2, 132.7, 156.4	115.8, 133.7, 162.7	128.0, 130.5, 160.9
Angles α, β, γ (°)	90, 90, 90	90, 90, 90	90, 100.7, 90
Wavelength (Å)	0.92	0.92	0.98
Resolution (Å)	46.6–2.90 (3.08–2.90)	49.1–3.20 (3.42–3.20)	48.8–3.55 (3.64–3.55)
R_{merge}	0.07 (1.09)	0.05 (0.72)	0.05 (0.74)
$R_{p.i.m.}$	0.04 (0.56)	0.05 (0.53)	0.04 (0.52)
$I/\sigma I$	22.8 (1.9)	14.9 (1.8)	12.8 (1.8)
CC1/2	0.999 (0.737)	0.999 (0.688)	0.999 (0.681)
Completeness (%)	99.3 (98.5)	98.8 (99.6)	99.3 (99.4)
Multiplicity	5.6 (5.3)	3.4 (3.5)	3.4 (3.4)
No. of unique reflections	26,924 (4,262)	20,889 (3,774)	62,574 (4,603)
Refinement Statistics			
Resolution	46.6–2.9	49.1–3.2	48.9–3.55
R_{work}/R_{free} (%)	21.7/26.4	22.6/27.3	22.3/26.5
No. of atoms			
Protein	6,093	6,053	24,413
Solvent	39	–	–
Ligand/ion	46	29	–
Average B factors (Å^2)			
All atoms	95	120	150
Protein	95	120	150
Solvent	80	–	–
Ligand/ion	150	140	–
Wilson B	85	110	130
RMSD			
Bond lengths (Å)	0.002	0.005	0.004
Bond angles (°)	0.533	1.1	1.0
Ramachandran plot			
Favored (%)	94.7	94.1	94.6
Allowed (%)	100	100	100
PDB	5AGA	5A9F	5A9J

Values in parentheses refer to the statistics in the highest resolution shell.

crystals reveals a modest difference in relative domain orientation (maximal displacements of up to 5 Å), which would place the ssDNA-binding motifs slightly closer together in the nucleotide-bound crystals (Figure S1B). Comparison with other homologs reveals that the D1-D2 conformation adopted by Polθ-HLD is most similar to the *A. fulgidus* Hel308 structure (Buttner et al., 2007), and distinct from either the *apo* or nucleotide-bound forms of the Hjm helicase from *P. furiosus* (Oyama et al., 2009), and the *S. solfataricus* Hel308 structure (Richards et al., 2008) (Figure S1C).

Structure of Domains 3 to 5

The C-terminal half of Polθ-HLD contains three additional globular domains that are not commonly found in SF2 helicases but are conserved across the Hel308 family. The first of these is a winged helix (WH) domain (residues 514–612), which is commonly found in DNA-binding proteins, including members of the RecQ family of helicases, where the wing of the WH is inserted at the interface of double-stranded DNA (dsDNA) and ssDNA, and forms a strand separation pin (Pike et al., 2009). WH domains are typically associated with binding to dsDNA by insertion of an α-helix (recognition helix) into the major groove and the wing contacting bases in the adjacent minor groove. In Polθ-HLD, the WH domain is quite closely associated with the D1 and D2 domains (Figure 2A) and, although the putative recognition helix and wing are exposed to solvent in such a way as to be accessible for DNA binding, no significant regions of aromatic or positively charged residues can be identified as would be required for DNA binding in the classical mode of other WH domains. Consistent with this, the WH domain of *A. fulgidus* Hel308 does not form any significant contacts to the bound DNA, thus the function of this domain within Polθ-HLD is unclear. Structural similarity searches using this domain have uncovered a surprisingly strong similarity (0.74 Å RMSD over 49 residues with 22%

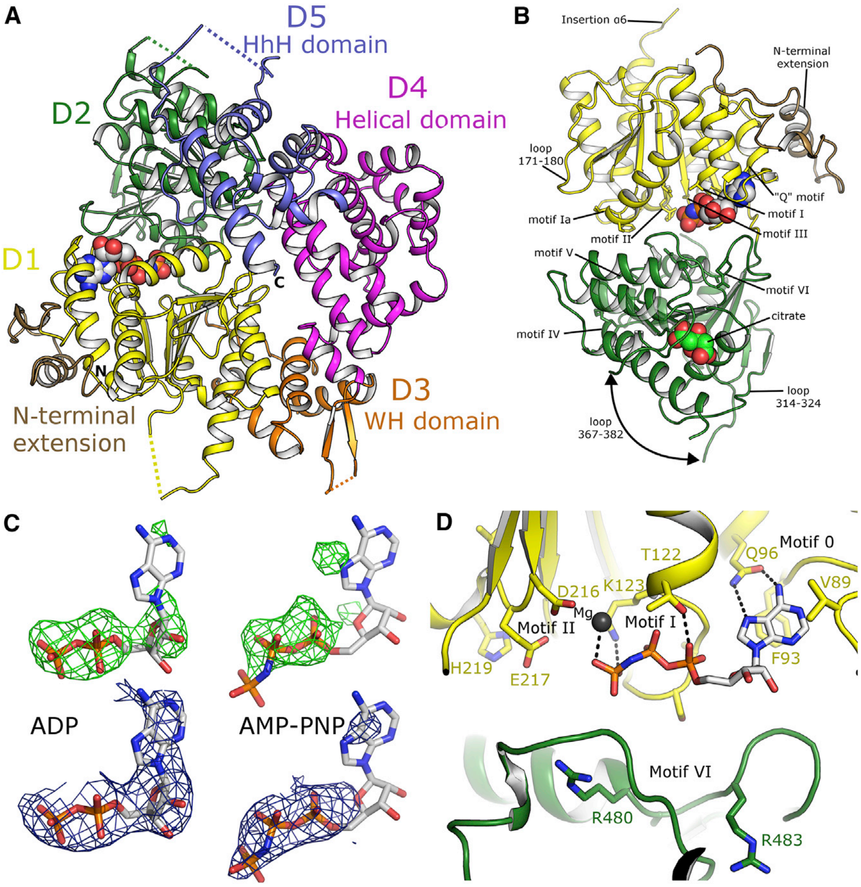

Figure 1. Structure of Polθ-HLD and ATPase Core

(A) Overview of the Polθ-HLD structure with domains colored individually (same color scheme is used throughout). The image is a composite of the AMP-PNP structure (PDB: 5AGA), with the nucleotide in the sphere representation, and the N-terminal extension from the *apo* crystals (PDB: 5A9J), which is colored brown. Dashed lines represent portions of the chains not well-ordered in the crystal structure.

(B) Close-up view of the core helicase domains D1 and D2, with the location of the conserved helicase motifs and variable regions labeled. The curved arrow shows the different path of loop 367–382, which is altered in the nucleotide-bound crystals, possibly because of the presence of a citrate ion (shown in sphere representation).

(C) Electron density maps covering the ADP and AMP-PNP nucleotides, the upper half shows $F_o - F_c$ omit maps colored green and contoured at 2.8σ, while the lower half shows the final refined $2F_o - 1F_c$ map contoured at 1.0σ in blue.

(D) Close-up view of the nucleotide-binding site with interacting residues labeled and polar contacts shown as dashed lines.

sequence identities) to the C-terminal WH domain of the 32-kDa subunit of replication protein A (RPA32c). In RPA32c, the WH domain serves as a protein interaction module with proteins involved in DNA damage response such as XPA, UNG, RAD52 TIPIN, and SMARCAL1 (Feldkamp et al., 2014), and a similar role in Polθ-HLD is a possibility, especially given the potential interaction interface is solvent exposed in the Polθ-HLD crystals.

Domain 4 in Polθ-HLD spans residues 613–789 and is all helical in character, containing 10 α-helices (we have used a slightly different nomenclature to the archaeal Hel308 proteins in which the first helix of this domain was considered part of the WH domain). The helical domain contacts the D2, WH, and domain 5, and in the Hel308 DNA complex structure this domain forms contacts to the DNA via three different areas (Buttner et al., 2007), with the C-terminal end of

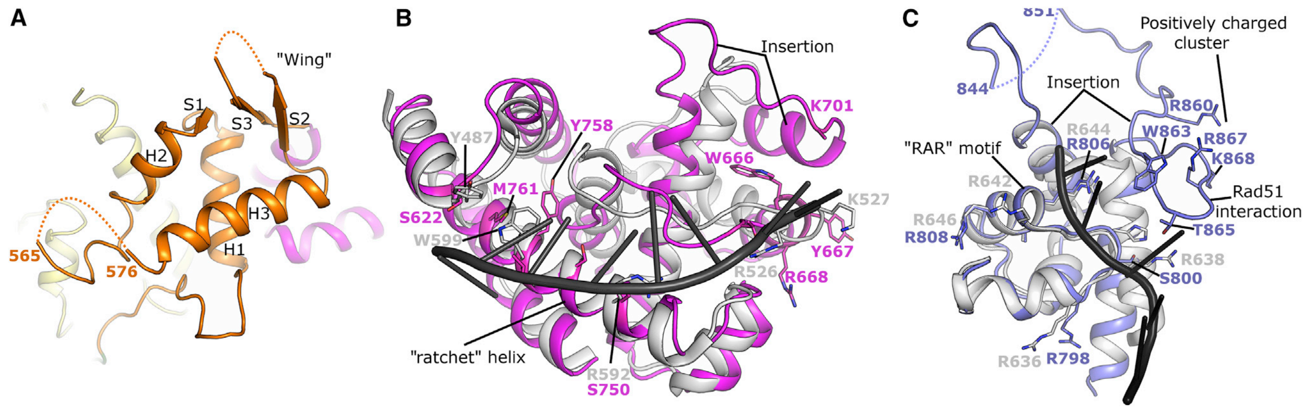

Figure 2. Structure and Comparison of Polθ-HLD Domains 3 to 5

(A) Close-up view of domain 3 (winged helix domain), with secondary structure elements labeled according to the canonical WH nomenclature. Dashed lines represent portions of the chains not well-ordered in the crystal structure.

(B) Close-up view of domain 4 (helical domain) with the equivalent domain (shown in gray) and DNA (shown in black) from *A. fulgidus* Hel308 shown for reference.

(C) Close-up view of domain 5 (helix-hairpin-helix domain) with the equivalent domain (shown in gray) and DNA (shown in black) from *A. fulgidus* Hel308 shown for reference.

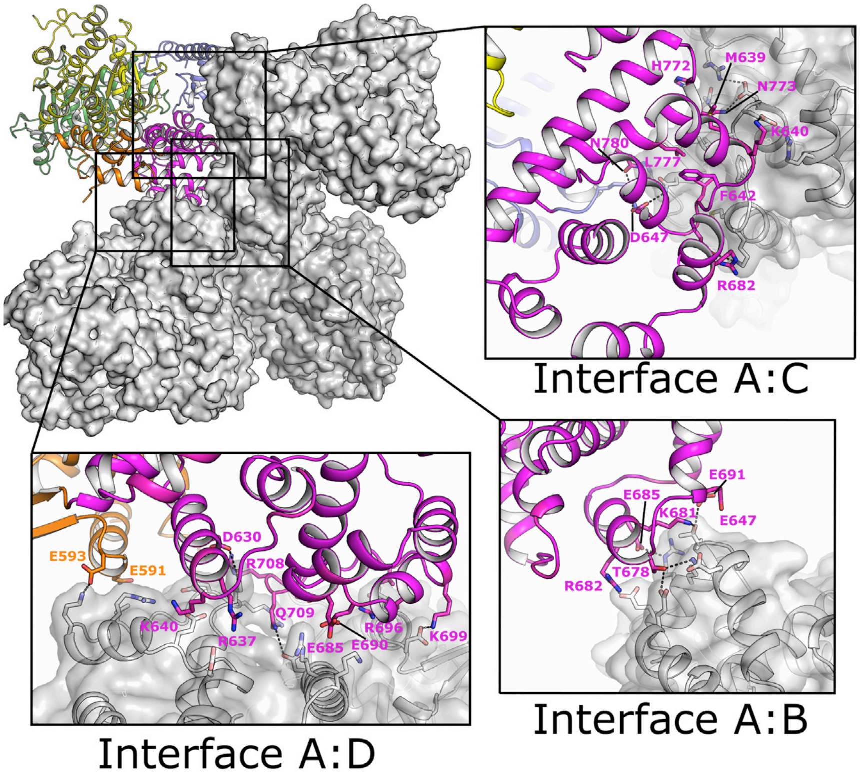

Figure 3. Structure of the Polθ-HLD Tetramer
Overview of the interfaces between chains in the Polθ-HLD crystals, with inset panels showing details of the protein contacts in each interface. Chain A is shown in backbone representation, and chains B–D are shown in surface representation.

the first helix providing van der Waals contacts to the single-stranded region, and two positively charged residues N-terminal at the end of the third helix, providing polar contacts to the DNA backbone at the double-stranded region. The most extensive area of interface comes from residues on the long curved ninth helix, which runs parallel to the path of the ssDNA and provides a mixture of polar and aromatic contacts primarily to the nucleobases of the DNA overhang. On the basis of this finding, this helix has been named the "ratchet helix" and suggested to function in ensuring directional transport of the DNA substrate. The equivalents of key DNA-binding residues in Polθ-HLD are generally not conserved (Figure 2B), although in many instances the substituted residues are consistent with DNA binding, suggesting that the specific nature of this domain's interaction with DNA will be considerably different. Another difference between Polθ-HLD domain 4 with the archaeal Hel308/Hjm structures is that significant insertions are present in the Polθ-HLD domain 4, most notably the region between the fourth and seventh helices, which in Polθ-HLD forms two additional α-helices and an extended loop. The insertions in domain 4 are in regions distant from the potential DNA contacts and appear to make contacts with neighboring molecules in the crystal (discussed later).

The final domain in Polθ-HLD (residues 790–891) contains a helix-hairpin-helix motif (HhH), in which is found proteins with diverse functions including DNA-binding activity (Doherty et al., 1996). Consistent with this, the equivalent domain in the Hel308 DNA complex interacts with the 3′ ssDNA tail, although in this complex the DNA binds to domain 5 in a looped-back manner, running in the opposite orientation to the rest of the molecule, leading the authors of this study to suggest that this domain may have a function in binding to a different DNA strand to the rest of the protein, possibly positioning Hel308 specifically onto the lagging strand of a replication fork (Buttner et al., 2007). Comparisons with Polθ-HLD reveal that, although a significant domain motion is required to superpose the two domains, the structures are well conserved at the potential DNA interface, including the "RAR" motif (Figure 2C). As is the case with domain 4, a significant insertion (38 residues) is evident in the Polθ-HLD structure, which forms an extended loop with a prominent β-hairpin that is partially disordered in both the nucleotide-bound and *apo* crystals (although to different extents). This prominent β-hairpin is almost entirely formed by one of the regions identified in a recent study to be responsible for binding Rad51 (Ceccaldi et al., 2015) (residues 861–868) which is at least partially solvent exposed. Three positively charged residues (R860, R867, and K868) from this region cluster together and form a significant positively charged feature on the protein surface, suggesting that electrostatic forces may play a significant role in the interaction (Figure 2C).

Quaternary Structure of Polθ-HLD

Analysis of the asymmetric unit of the *apo* crystals reveals four forming a tetrameric clover-shaped molecule with D2 symmetry (Figure 3). Analysis of the nucleotide-bound crystals reveals that the same arrangement can be formed from the crystallographic symmetry operators, suggesting the tetramer is unlikely to be a result of crystal packing interactions. Each subunit in the potential tetramer contacts all three of its neighbors (Figure 3A), with the interface between chains AC and BD in the *apo* crystals being slightly more extensive (850-Å^2 interface area) and hydrophobic than the interface linking chains AD and BC (600-Å^2 interface area), as calculated by the program PISA (Krissinel and Henrick, 2007). Finally, a much smaller interface (180 Å^2) that is composed entirely of polar contacts connects AB and CD. The interfaces are almost entirely composed of contacts from the inserted regions of domain 4, explaining why the quaternary structure is not generally conserved across the Hel308 family (archaeal Hel308/Hjm proteins were reported to be monomeric). Furthermore, the fact that the D1 and D2 domains are not constrained by symmetry contacts suggests they would be free to move relative to each other as is required for the helicase mechanism. Higher-order quaternary structures are not unprecedented in SF2 helicases, for example, the human RECQ1 helicase has a

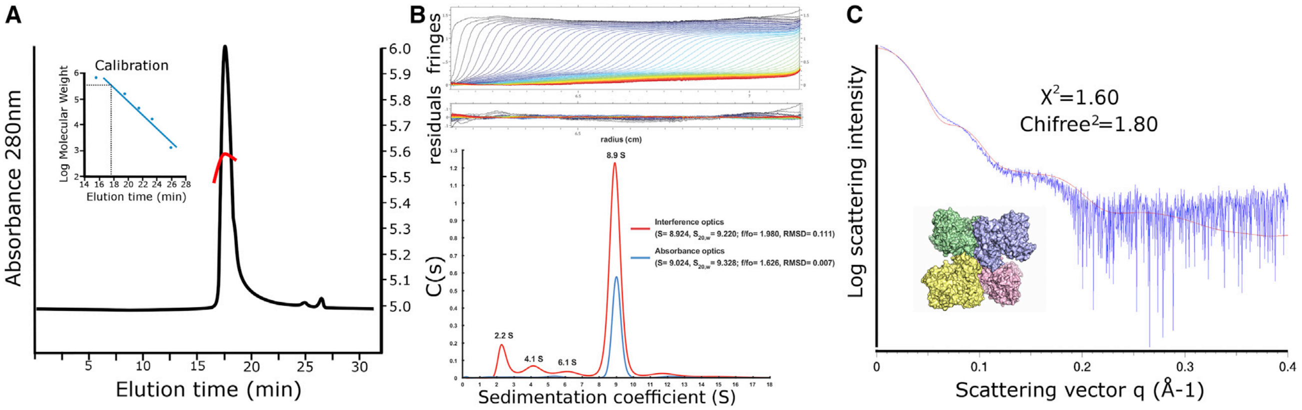

Figure 4. Analysis of Polθ-HLD in Solution

(A) Analysis of Polθ-HLD in solution by SEC-MALS. The main plot shows a chromatogram of Polθ-HLD on an analytical gel filtration column with the calibration plot shown in the insert. The red curve shows the distribution of molecular masses (right hand *y*-axis) calculated by MALS.

(B) Analysis of Polθ-HLD (amino acids 67–894) by sedimentation velocity AUC. Upper and middle panels show data and residuals plots for interference scans, with the lower panels showing sedimentation coefficient distribution plots for both interference and absorbance scans.

(C) Analysis of Polθ-HLD in solution by SAXS. The experimental data (blue) are plotted as a function of scattering vector q, with the theoretical scattering curve calculated from the PolQ tetramer (shown in the surface representation) overlaid in red.

tetrameric structure, which is essential for activity on Holliday junctions but not on simpler fork-like substrates (Pike et al., 2015).

Solution Studies of Polθ-HLD

We have performed a number of solution measurements to further analyze the quaternary structure of Polθ-HLD in solution. Analysis of Polθ-HLD by size-exclusion chromatography with multi-angle light scattering (SEC-MALS) reveals a single species with an elution volume consistent with a particle with molecular mass of 360 kDa and light-scattering profiles consistent with a particle of 379 kDa, both almost exactly four times the monomer mass (93,080 Da) (Figure 4A). Consistent with this, sedimentation velocity analytical ultracentrifugation (AUC) of the same Polθ-HLD construct suggested a major species with a sedimentation coefficient of around 8.9 ($S_{20,w}$ = 9.2–9.3) (Figure 4B). This is consistent with a particle mass of four times the monomer mass for inference optics, and 3.2 times with absorbance. Smaller species suggestive of monomer, dimer, or degradation products were present, but at very small fractions compared with the suggested tetramer. The relatively large frictional ratio suggests a significantly more elongated or flexible protein in solution than that appearing in the crystal structure, which may account for the difference between the observed and the theoretical sedimentation coefficients ($S_{20,w}$ = 11.6) calculated from the Polθ-HLD tetramer X-ray crystal structure using the program HYDROPRO (Ortega et al., 2011). We have also performed an analysis of Polθ-HLD in solution using small-angle X-ray scattering (SAXS). Scattering profiles of Polθ-HLD show radius of gyration values of around 52 Å (calculated in both real and reciprocal space) and volumes of correlation consistent with a large oligomer with an approximate mass of 390 kDa. The experimental scattering profiles show distinctive humps at q values of 0.04, 0.09, and 0.15 $Å^{-1}$ (Figure 4C). Comparing these profiles directly with theoretical scattering profiles calculated from the Polθ-HLD tetramer crystal structure reveals good agreement between the experimental profiles and the Polθ-HLD tetramer in crystals (χ^2 = 1.60, χ^2 free = 1.80) (Figure 3D). The SAXS data shown in Figure 4C were collected on the 67–894 construct in the presence of 1 mM ADP Mg^{2+}, while the SEC-MALS and AUC data were collected on the same construct in the absence of nucleotide, suggesting the tetramer in solution is present irrespective of the nucleotide-binding status of Polθ-HLD.

Characterization of Polθ-HLD DNA-Binding and ATPase Activity

Previous studies of Polθ-HLD have shown qualitatively that Polθ-HLD has DNA-binding and DNA-stimulated ATPase activity but have so far failed to demonstrate any helicase activity (Ceccaldi et al., 2015; Seki et al., 2003). We performed a more quantitative characterization of the DNA-binding activity using a fluorescence polarization-based assay against a variety of DNA substrates generated using a similar approach to that used for characterization of UvrD helicase (Sharma and Rao, 2012) (Tables S1A and S1B). We noticed that the apparent dissociation constant obtained was significantly affected by the salt concentration in the assay buffer, as is common for DNA-binding proteins, with an apparent 10-fold tighter dissociation constant (single digit nM) obtained for ssDNA in low salt buffer (10 mM HEPES [pH 7.5], 50 mM NaCl) (Figure S3). Fluorescence polarization assays, performed in a near-physiological ionic-strength buffer (10 mM HEPES [pH 7.5], 150 mM NaCl) (Figure 5A) show a general trend of Polθ-HLD binding to substrates with significant regions of ssDNA binding relatively tightly (k_D = 20–50 nM), complex substrates with only dsDNA (three- and four-way junctions) being somewhat intermediate (k_D = 60–90 nM), and dsDNA showing significantly weaker binding (k_D = 190 nM). Given the sensitivity of the assay technique, it is not clear if the modest differences between similar substrates are significant.

We have also performed helicase assays using radiolabeled versions of the same substrates. As was the case for a number

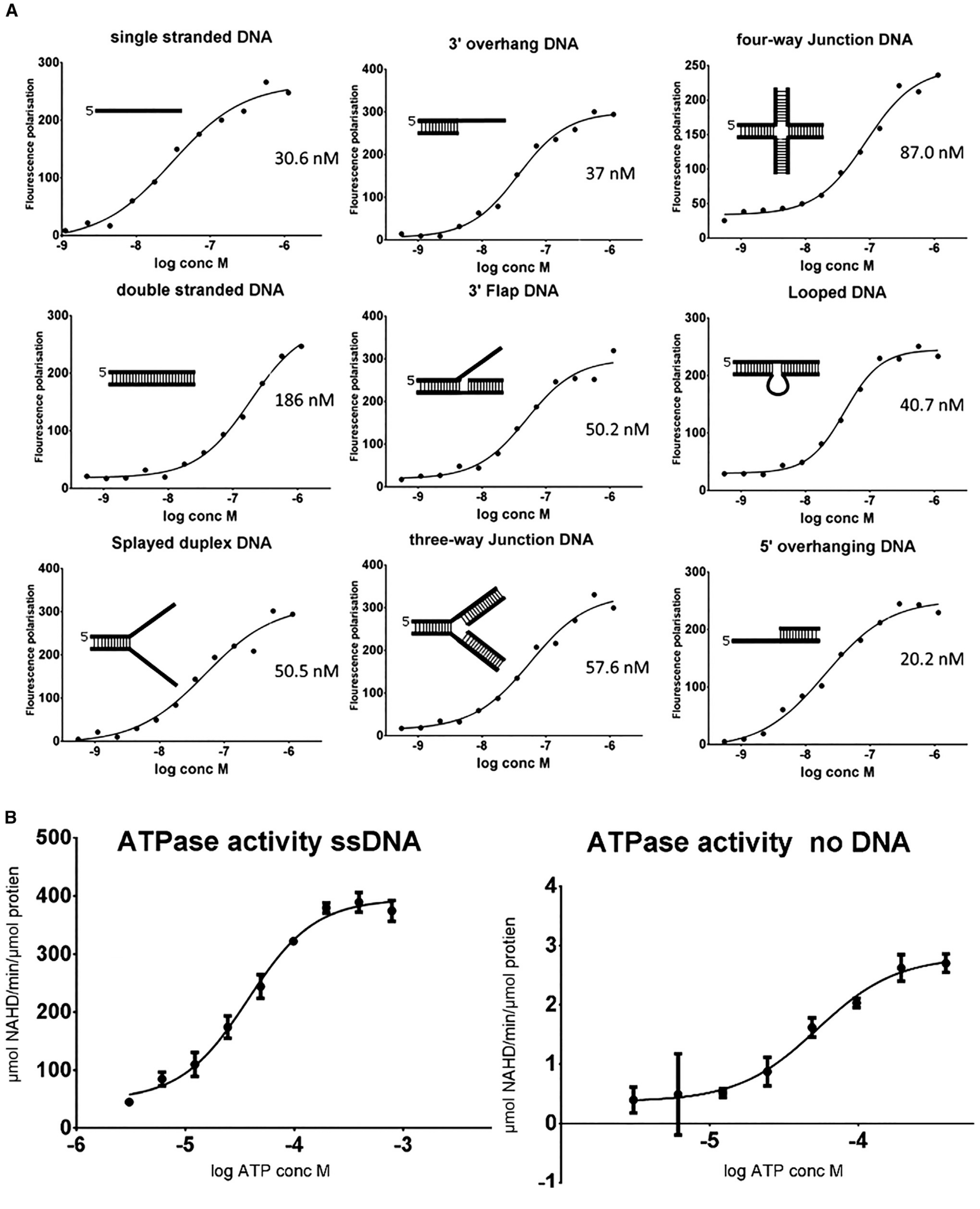

Figure 5. Characterization of DNA-Binding and ATPase Activity of Polθ-HLD

(A) Fluorescence polarization DNA-binding assays with various DNA substrates (sequences in Table S1).

(B) ATPase activity of Polθ-HLD with and without stimulation by single-stranded DNA. Error bars are plotted ± standard error of at least three independent replicates.

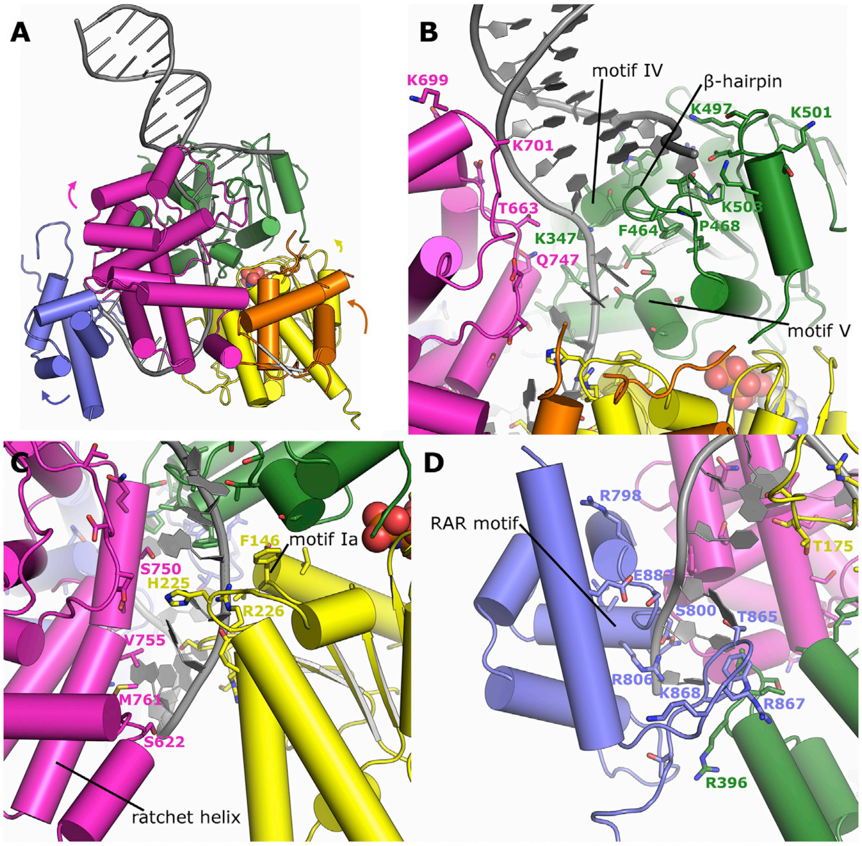

Figure 6. Modeling of the Interaction Between Polθ-HLD and DNA

(A) Overview of the Polθ-HLD DNA complex model, with the DNA shown in gray. The colored arrows show the relative movements applied to the corresponding domains.

(B) Close-up view of the Polθ-HLD DNA complex model at the junction between double- and single-stranded regions, with key conserved ssDNA-binding residues and motifs labeled.

(C) View of the Polθ-HLD DNA complex model around the interface between the ssDNA and Polθ-HLD D2/D4, with key DNA-binding residues and motifs labeled.

(D) View of the Polθ-HLD DNA complex model around the interface between the 3′ ssDNA end and the HhH domain (D5), with key DNA-binding residues and motifs labeled.

of previous investigations of Polθ-HLD (Maga et al., 2002; Seki et al., 2003), we were unable to show any helicase activity, although we were able to demonstrate a significant stimulation of ATPase activity by ssDNA. The K_m for ATP is similar in the presence or absence of DNA (40–50 μM; Figure 5B), but the K_{cat} is increased 140-fold (to ~400 min^{-1}). These kinetic parameters are quite typical when compared with other helicases of a similar type and indicate that the reason for lack of observed helicase activity in Polθ-HLD is not due to a defect in the core ATPase machinery.

Model of Polθ-HLD Bound to DNA

To gain further insight into the interaction of Polθ-HLD with DNA, we have used the Hel308 DNA complex (Buttner et al., 2007) to construct a model of Polθ-HLD bound to DNA containing an extended 3′ overhang. As is the case with previous modeling studies (Oyama et al., 2009), a global superposition reveals Polθ-HLD is not quite in an overall conformation consistent with DNA binding, and relative domain motions are necessary to avoid steric clashes. For this reason, we have created a hybrid model in which the five domains of Polθ-HLD are individually superposed onto the Hel308 DNA complex (Figure 6A). In this model, the majority of the steric clashes are removed, although a number of additional side-chain and nucleotide movements would be required to fully accommodate the DNA. As is the case in the Hel308 DNA complex, the phosphodiester backbone of the final 3–4 bp of the dsDNA is in position to make several polar contacts to residues in both the D2 domain (K347, K348, K352, and K497) and domain 4 (T663, Y667, K699, and K701). Several of the contacts to domain 4 are from residues in inserted regions with respect to the archaeal Hel308/Hjm proteins, suggesting these regions also contribute to DNA binding. Another feature that differs between Polθ-HLD and other Hel308 family helicases is the extent of the β-hairpin, which in Polθ-HLD is significantly shorter and lacks equivalents of the aromatic residues that form interactions with the final paired bases in the Hel308 DNA complex structure (Figures 6B and S4). The 3′ overhang then passes through a tight cavity created primarily by D1, D2, and domain 4, with the first three phosphates of the overhang contacting conserved residues in the D2 domain (helicase motifs IV and V), and the fourth to sixth phosphates contacting the D1 domain (helicase motifs Ia and Ib). The nucleobases face primarily toward domain 4 and are in positions with the potential to form a number of polar contacts to nearby side-chain residues. The potential ratcheting mechanism that has been suggested to be a feature of the *A. fulgidus* Hel308 mechanism is not conserved in Polθ-HLD, although it is possible that the bulky hydrophobic residues of V757 and M761 may provide a similar function creating a barrier resistant to backward movement of the DNA (Figures 2B and 6C). The final six nucleotides at the 3′ ends exit the cavity, and in the Hel308 DNA complex loop back with the final three nucleotides binding to domain 5 in the opposite orientation to the rest of the overhang. This mode of binding is a possibility for Polθ-HLD, although significant shifts were required to position this domain, and even after this shift some minor steric clashes remain (Figure 6D).

Extrapolating the Polθ-HLD DNA complex model onto the Polθ-HLD tetramer reveals significant insights into a possible role of Polθ-HLD function in DNA repair pathways. The symmetry between chains AC and BD is such that, if both subunits are bound to DNA simultaneously, the two 3′ ends exit from the cavity in close proximity and point in opposite directions (Figure 7A). Continuing the path of these two strands leads to a number of possibilities, for example the 3′ end of the DNA to become

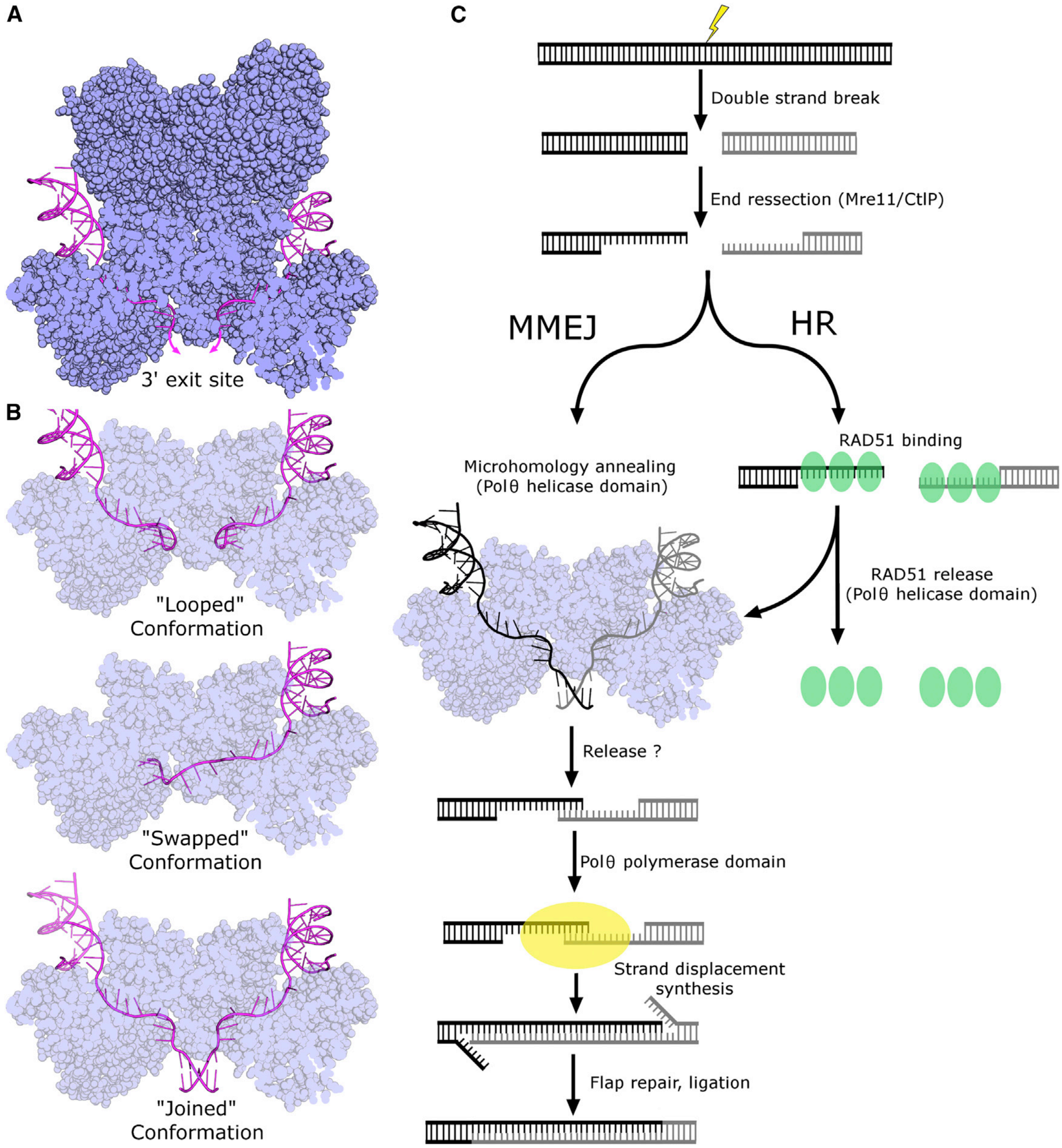

Figure 7. Possible Modes of DNA Binding to the Polθ-HLD Tetramer, and their Implications for the MMEJ Pathway

(A) Surface representation of the Polθ-HLD tetramer, with DNA substrates modeled into the bottom two subunits. The 3′ ends of the DNA strands exit the main DNA-binding cavity in close proximity.

(B) Models of alternative modes for DNA binding (only the bottom half of the tetramer shown for clarity). The "Looped" conformation is observed in the Hel308 DNA complex structure where the 3′ ends bind in a reverse orientation to domain 5. The "Swapped" conformation may occur if the 3′ end from one molecule is able to associate with domain 5 of a neighboring molecule. The "Joined" conformation may occur if some small region (~5 bp) of microhomology exists between the two strands.

(C) Both MMEJ and HR double-strand break repair pathways share (and compete for) the initial end resection step. Polθ-HLD, in addition to its anti-recombinase activity, may be able to catalyze the "microhomology annealing" step and present the annealed substrate for subsequent processing by the Polθ polymerase domain.

bound to domain 5 of the neighboring subunit in a "swapped" rather than the "looped" conformation adopted by the Hel308 DNA complex (Figure 7B). We have also considered the possible implications of these two emerging strands with respect to the polymerase activity of the C-terminal polymerase domain. If a small area of sequence complementarity exists between them, their proximity and positioning may encourage the formation of short sections of dsDNA (Figure 7B). This would then be the preferred substrate (containing both template strand and a 3′ hydroxyl primer) for the polymerase domain to bind and initiate synthesis. If the two strands brought together by the Polθ-HLD were the result of a double-strand break that has undergone end

resection by Mre11 or CtIP nucleases (Truong et al., 2013), the close positioning of these two strands by the helicase domain and subsequent joining by the polymerase domain would be a plausible model of how the polymerase and helicase domains of Polθ are able to repair double-strand breaks by the MMEJ pathway (Figure 7C). We have attempted to develop an assay to test this activity in vitro, using DNA crosslinking and FRET-based assays to capture the possibly transient annealing of DNA strands containing only short (6 bp or less) microhomologies; however, we are unable to demonstrate this activity experimentally, possibly for technical reasons.

DISCUSSION

In this study we have determined the structure of Polθ-HLD. The structure displays a number of deep cavities and pockets, some of which are sites of nucleotide or DNA binding, and some created by the arrangement of subunits in the Polθ-HLD tetramer. It is conceivable that these cavities may represent "druggable" sites for the development of Polθ-HLD inhibitors that could be used to specifically damage certain types of cancer cells deficient in HR (Ceccaldi et al., 2015; Higgins et al., 2010a). Comparisons with other helicases allow us to identify a number of unique features based on inserted regions, the most significant of which are additional helices of domain 4 that contribute to an extensive tetramer interface that we have shown to be stable in solution. It has previously been speculated that multimerization of the polymerase domain of Polθ may be a requirement for some of its enzymatic activities (Kent et al., 2015; Zahn et al., 2015), and although the polymerase domain behaves primarily as a monomer on size-exclusion chromatography (Kent et al., 2015; Zahn et al., 2015), two potential dimer interfaces were identified in the crystal structure of the polymerase domain (Zahn et al., 2015). We note that it is possible in the context of the full-length protein for these symmetry elements to be combined, with each half of a single helicase domain tetramer connected to a dimer of the polymerase domain. The tetrameric arrangement of the helicase domain may also explain how Polθ is able to promote chromosomal translocations (Mateos-Gomez et al., 2015), with the possibility of binding to, and promoting exchanges with, two distinct pairs of double-strand breaks within the same molecule.

Our enzymatic characterization of Polθ-HLD showed relatively strong ssDNA-binding activity for various substrates, and a robust DNA-stimulated ATPase activity that is comparable with other helicases. In common with previous studies of Polθ-HLD, we were unable to demonstrate any helicase activity. It is possible that this is due to the requirement for a specific substrate, although this is not generally the case for other helicases assayed in vitro with specialized functions.

We have modeled DNA in the Polθ-HLD structure using the DNA from the *A. fulgidus* Hel308 DNA complex. We did not see any single obvious feature that would prevent Polθ-HLD from functioning as a helicase, although the relatively short β-hairpin (Figure S3) and lack of conserved contacts in the ratchet helix indicate that the defect, if present, most likely lies in the ability to couple ATP hydrolysis to directional movement along the DNA. This is consistent with the fact that no global conformational changes were observed between the ADP- and AMP-PNP-bound states, and only relatively modest changes were observed when the nucleotide-bound states were compared with the *apo* state, This is similar to the situation observed in the Swi/Snf chromatin remodeling factors such as Rad54 (Thoma et al., 2005), which also lack helicase activity, yet the only significant difference that could be observed relative to related active helicases is the lack of an effective DNA unwinding wedge (Thoma et al., 2005).

We have also noticed that the symmetry of Polθ-HLD would place the emerging ends of the 3′ overhangs in close proximity, and if short sections (4–6 bp) of complementarity existed between them, it is possible that one of the functions of the helicase domain could be to catalyze this strand-annealing step. In this context, the lack of helicase activity would begin to make sense as significant processive helicase activity would have the potential to introduce large mutagenic deletions to the repair pathway. The DNA substrate used in our modeling studies (14 nucleotides 3′ overhang with five bases of homology at the 3′ terminus) is similar to structures produced by Mre11/CtIP (Cannavo and Cejka, 2014) (15–20 nucleotides in yeast) and the preferred substrate for the polymerase domain (4–6 bp of homology with an overhang <18 bp) (Kent et al., 2015). While the Polθ polymerase domain alone has recently been shown to catalyze both the strand-annealing and overhang extension stages of MMEJ in vitro (Kent et al., 2015), the demonstration of anti-recombinase activity linked specifically to the helicase domain (Ceccaldi et al., 2015) means that in vivo, the competing presence of a number of additional ssDNA-binding factors, for example RPA and RAD51, would make the first step relatively inefficient. The fact that the initial stages of both HR and MMEJ share the same substrate would place the two pathways in competition for the same cellular resource. In this context, the joining of a helicase with dedicated roles in displacing HR intermediates and DNA strand annealing to a polymerase capable of performing overhang extension from poorly annealed templates would be an elegant solution to the problem of double-strand break repair.

EXPERIMENTAL PROCEDURES

Protein Expression and Purification

The Polθ-HLD constructs (residues 1–894 and 67–894) were expressed with a His_6 tag in baculovirus-infected insect cells. The proteins were purified by immobilized metal affinity chromatography, cleavage of the tag, and gel filtration.

Crystallization and Structure Determination

Crystals of AMP-PNP- and ADP-bound Polθ-HLD were obtained at 20°C from solutions containing 19% PEG 3350, 0.2 M potassium citrate tribasic (pH 8.5), 10 mM $MgCl_2$, and 10 mM of either nucleotide, and the *apo* protein crystallized in 0.2 M NaCl, 0.1 M HEPES (pH 7.0), 20% PEG 6K, and 10% ethylene glycol. The structures were solved by molecular replacement with a *P. furiosis* Hjm (Oyama et al., 2009) structure as a starting model. The structures were deposited in the PDB (PDB: 5A9J, 5AGA, 5A9F).

Analytical Size-Exclusion Chromatography

Analytical online SEC-MALS was performed on a Viscotek TDA 305 system (Malvern Instruments) using a 15-ml Shodex KW-803 silica column (GE Healthcare).

Analytical Ultracentrifugation

Sedimentation velocity AUC experiments were performed on an XL-I Analytical Ultracentrifuge (Beckman Coulter). Absorbance (280 nm) and interference data were analyzed with SEDFIT (Schuck, 2000) and SEDNTERP (Harding

et al., 1992). Theoretical sedimentation coefficients of model proteins were calculated from Polθ-HLD structures using the program HYDROPRO (Ortega et al., 2011).

Small-Angle X-Ray Scattering

SAXS measurements of Polθ-HLD in solution were performed at Diamond Light Source beamline B21 using a BIOSAXS robot for sample loading. The data were reduced and buffer contributions subtracted with the DawnDiamond software suite, and analyzed using the program SCATTER (www.bioisis.net). Real-space scattering profiles of atomic models were calculated from atomic models using CRYSOL (Svergun et al., 1995) and aligned and scaled to the experimental data using PRIMUS (Konarev et al., 2003). The agreement between theoretical and experimental scattering profiles was evaluated using the χ^2-free procedure (Rambo and Tainer, 2013) implemented in the program SCATTER.

DNA-Binding Assays

DNA binding was measured using fluorescence polarization. Using the oligonucleotides listed in Table S1, kinetic constants were calculated from binding curves with a four-parameter logarithmic binding equation using the program PRISM (GraphPad).

ATPase Activity Assays

ATPase activity of Polθ-HLD was measured using a pyruvate kinase, lactate dehydrogenase enzyme-linked absorbance assay (Norby, 1988) with ATP concentrations between 3 μM and 0.8 mM. For DNA-stimulated ATPase, the reaction mix contained in addition 2 μM of single-stranded 18-bp DNA.

SUPPLEMENTAL INFORMATION

Supplemental Information includes Supplemental Experimental Procedures, four figures, and one table and can be found with this article online at http://dx.doi.org/10.1016/j.str.2015.10.014.

AUTHOR CONTRIBUTIONS

J.A.N. performed the biochemical experiments, and crystallized and solved the crystal structures; C.D.O.C. designed and executed cloning and protein expression, biochemical experiments and protein crystallization; H.A. purified the proteins and performed the assays; J.A.N., C.D.O.C., and O.G. wrote the manuscript.

ACKNOWLEDGMENTS

We thank James Douch for help with SAXS data collection and analysis, Katarzyna Kupinska and Nicola Burgess-Brown for baculovirus support, and Dr. David Staunton of the Oxford University Biophysics Facility (Biochemistry Department) for assistance with AUC. We acknowledge Diamond Light Source for time on beamlines I02, I03, I04-1, I24, and B21 under Proposals MX8421 and MX10619. The SGC is a registered charity (number 1097737) that receives funds from AbbVie, Bayer Pharma AG, Boehringer Ingelheim, the Canada Foundation for Innovation, Genome Canada, GlaxoSmithKline, Janssen, Lilly Canada, Merck & Co., the Novartis Research Foundation, the Ontario Ministry of Economic Development and Innovation, Pfizer, São Paulo Research Foundation-FAPESP, Takeda, and the Wellcome Trust [092809/Z/10/Z]. C.D.O.C. thanks the E. P. Abraham Cephalosporin Fund and Linacre College for support.

Received: July 28, 2015
Revised: October 6, 2015
Accepted: October 9, 2015
Published: December 1, 2015

REFERENCES

Arana, M.E., Seki, M., Wood, R.D., Rogozin, I.B., and Kunkel, T.A. (2008). Low-fidelity DNA synthesis by human DNA polymerase theta. Nucleic Acids Res. *36*, 3847–3856.

Bernstein, D.A., Zittel, M.C., and Keck, J.L. (2003). High-resolution structure of the E.coli RecQ helicase catalytic core. EMBO J. *22*, 4910–4921.

Braithwaite, D.K., and Ito, J. (1993). Compilation, alignment, and phylogenetic relationships of DNA polymerases. Nucleic Acids Res. *21*, 787–802.

Buttner, K., Nehring, S., and Hopfner, K.P. (2007). Structural basis for DNA duplex separation by a superfamily-2 helicase. Nat. Struct. Mol. Biol. *14*, 647–652.

Cancer Genome Atlas Research Network. (2011). Integrated genomic analyses of ovarian carcinoma. Nature *474*, 609–615.

Cannavo, E., and Cejka, P. (2014). Sae2 promotes dsDNA endonuclease activity within Mre11-Rad50-Xrs2 to resect DNA breaks. Nature *514*, 122–125.

Ceccaldi, R., Liu, J.C., Amunugama, R., Hajdu, I., Primack, B., Petalcorin, M.I., O'Connor, K.W., Konstantinopoulos, P.A., Elledge, S.J., Boulton, S.J., et al. (2015). Homologous-recombination-deficient tumours are dependent on Poltheta-mediated repair. Nature *518*, 258–262.

Doherty, A.J., Serpell, L.C., and Ponting, C.P. (1996). The helix-hairpin-helix DNA-binding motif: a structural basis for non-sequence-specific recognition of DNA. Nucleic Acids Res. *24*, 2488–2497.

Feldkamp, M.D., Mason, A.C., Eichman, B.F., and Chazin, W.J. (2014). Structural analysis of replication protein A recruitment of the DNA damage response protein SMARCAL1. Biochemistry *53*, 3052–3061.

Goff, J.P., Shields, D.S., Seki, M., Choi, S., Epperly, M.W., Dixon, T., Wang, H., Bakkenist, C.J., Dertinger, S.D., Torous, D.K., et al. (2009). Lack of DNA polymerase theta (POLQ) radiosensitizes bone marrow stromal cells in vitro and increases reticulocyte micronuclei after total-body irradiation. Radiat. Res. *172*, 165–174.

Guy, C.P., and Bolt, E.L. (2005). Archaeal Hel308 helicase targets replication forks in vivo and in vitro and unwinds lagging strands. Nucleic Acids Res. *33*, 3678–3690.

Gyimesi, M., Sarlos, K., and Kovacs, M. (2010). Processive translocation mechanism of the human Bloom's syndrome helicase along single-stranded DNA. Nucleic Acids Res. *38*, 4404–4414.

Harding, S.E., Rowe, A.J., and Horton, J.C. (1992). Analytical Ultracentrifugation in Biochemistry and Polymer Science (Royal Society of Chemistry).

Higgins, G.S., Harris, A.L., Prevo, R., Helleday, T., McKenna, W.G., and Buffa, F.M. (2010a). Overexpression of POLQ confers a poor prognosis in early breast cancer patients. Oncotarget *1*, 175–184.

Higgins, G.S., Prevo, R., Lee, Y.F., Helleday, T., Muschel, R.J., Taylor, S., Yoshimura, M., Hickson, I.D., Bernhard, E.J., and McKenna, W.G. (2010b). A small interfering RNA screen of genes involved in DNA repair identifies tumor-specific radiosensitization by POLQ knockdown. Cancer Res. *70*, 2984–2993.

Hogg, M., Seki, M., Wood, R.D., Doublie, S., and Wallace, S.S. (2011). Lesion bypass activity of DNA polymerase theta (POLQ) is an intrinsic property of the pol domain and depends on unique sequence inserts. J. Mol. Biol. *405*, 642–652.

Hogg, M., Sauer-Eriksson, A.E., and Johansson, E. (2012). Promiscuous DNA synthesis by human DNA polymerase theta. Nucleic Acids Res. *40*, 2611–2622.

Inagaki, S., Suzuki, T., Ohto, M.A., Urawa, H., Horiuchi, T., Nakamura, K., and Morikami, A. (2006). Arabidopsis TEBICHI, with helicase and DNA polymerase domains, is required for regulated cell division and differentiation in meristems. Plant Cell *18*, 879–892.

Kawamura, K., Bahar, R., Seimiya, M., Chiyo, M., Wada, A., Okada, S., Hatano, M., Tokuhisa, T., Kimura, H., Watanabe, S., et al. (2004). DNA polymerase theta is preferentially expressed in lymphoid tissues and upregulated in human cancers. Int. J. Cancer *109*, 9–16.

Kent, T., Chandramouly, G., McDevitt, S.M., Ozdemir, A.Y., and Pomerantz, R.T. (2015). Mechanism of microhomology-mediated end-joining promoted by human DNA polymerase theta. Nat. Struct. Mol. Biol. *22*, 230–237.

Konarev, P.V., Volkov, V.V., Sokolova, A.V., Koch, M.H.J., and Svergun, D.I. (2003). PRIMUS: a windows PC-based system for small-angle scattering data analysis. J. Appl. Crystallogr. *36*, 1277–1282.

Krissinel, E., and Henrick, K. (2007). Inference of macromolecular assemblies from crystalline state. J. Mol. Biol. *372*, 774–797.

Lemee, F., Bergoglio, V., Fernandez-Vidal, A., Machado-Silva, A., Pillaire, M.J., Bieth, A., Gentil, C., Baker, L., Martin, A.L., Leduc, C., et al. (2010). DNA polymerase theta up-regulation is associated with poor survival in breast cancer, perturbs DNA replication, and promotes genetic instability. Proc. Natl. Acad. Sci. USA *107*, 13390–13395.

Maga, G., Shevelev, I., Ramadan, K., Spadari, S., and Hubscher, U. (2002). DNA polymerase theta purified from human cells is a high-fidelity enzyme. J. Mol. Biol. *319*, 359–369.

Mateos-Gomez, P.A., Gong, F., Nair, N., Miller, K.M., Lazzerini-Denchi, E., and Sfeir, A. (2015). Mammalian polymerase theta promotes alternative NHEJ and suppresses recombination. Nature *518*, 254–257.

McVey, M., and Lee, S.E. (2008). MMEJ repair of double-strand breaks (director's cut): deleted sequences and alternative endings. Trends Genet. *24*, 529–538.

Norby, J.G. (1988). Coupled assay of Na+,K+-Atpase activity. Method Enzymol. *156*, 116–119.

Ortega, A., Amoros, D., and Garcia de la Torre, J. (2011). Prediction of hydrodynamic and other solution properties of rigid proteins from atomic- and residue-level models. Biophys. J. *101*, 892–898.

Oyama, T., Oka, H., Mayanagi, K., Shirai, T., Matoba, K., Fujikane, R., Ishino, Y., and Morikawa, K. (2009). Atomic structures and functional implications of the archaeal RecQ-like helicase Hjm. BMC Struct. Biol. *9*, 2.

Pike, A.C., Shrestha, B., Popuri, V., Burgess-Brown, N., Muzzolini, L., Costantini, S., Vindigni, A., and Gileadi, O. (2009). Structure of the human RECQ1 helicase reveals a putative strand-separation pin. Proc. Natl. Acad. Sci. USA *106*, 1039–1044.

Pike, A.C., Gomathinayagam, S., Swuec, P., Berti, M., Zhang, Y., Schnecke, C., Marino, F., von Delft, F., Renault, L., Costa, A., et al. (2015). Human RECQ1 helicase-driven DNA unwinding, annealing, and branch migration: insights from DNA complex structures. Proc. Natl. Acad. Sci. USA *112*, 4286–4291.

Prasad, R., Longley, M.J., Sharief, F.S., Hou, E.W., Copeland, W.C., and Wilson, S.H. (2009). Human DNA polymerase theta possesses 5'-dRP lyase activity and functions in single-nucleotide base excision repair in vitro. Nucleic Acids Res. *37*, 1868–1877.

Rambo, R.P., and Tainer, J.A. (2013). Accurate assessment of mass, models and resolution by small-angle scattering. Nature *496*, 477–481.

Richards, J.D., Johnson, K.A., Liu, H., McRobbie, A.M., McMahon, S., Oke, M., Carter, L., Naismith, J.H., and White, M.F. (2008). Structure of the DNA repair helicase hel308 reveals DNA binding and autoinhibitory domains. J. Biol. Chem. *283*, 5118–5126.

Schuck, P. (2000). Size-distribution analysis of macromolecules by sedimentation velocity ultracentrifugation and lamm equation modeling. Biophys. J. *78*, 1606–1619.

Seki, M., and Wood, R.D. (2008). DNA polymerase theta (POLQ) can extend from mismatches and from bases opposite a (6-4) photoproduct. DNA Repair (Amst.) *7*, 119–127.

Seki, M., Marini, F., and Wood, R.D. (2003). POLQ (Pol theta), a DNA polymerase and DNA-dependent ATPase in human cells. Nucleic Acids Res. *31*, 6117–6126.

Seki, M., Masutani, C., Yang, L.W., Schuffert, A., Iwai, S., Bahar, I., and Wood, R.D. (2004). High-efficiency bypass of DNA damage by human DNA polymerase Q. EMBO J. *23*, 4484–4494.

Shaheen, M., Allen, C., Nickoloff, J.A., and Hromas, R. (2011). Synthetic lethality: exploiting the addiction of cancer to DNA repair. Blood *117*, 6074–6082.

Sharief, F.S., Vojta, P.J., Ropp, P.A., and Copeland, W.C. (1999). Cloning and chromosomal mapping of the human DNA polymerase theta (POLQ), the eighth human DNA polymerase. Genomics *59*, 90–96.

Sharma, R., and Rao, D.N. (2012). Functional characterization of UvrD helicases from Haemophilus influenzae and Helicobacter pylori. FEBS J. *279*, 2134–2155.

Shima, N., Hartford, S.A., Duffy, T., Wilson, L.A., Schimenti, K.J., and Schimenti, J.C. (2003). Phenotype-based identification of mouse chromosome instability mutants. Genetics *163*, 1031–1040.

Singleton, M.R., Dillingham, M.S., and Wigley, D.B. (2007). Structure and mechanism of helicases and nucleic acid translocases. Annu. Rev. Biochem. *76*, 23–50.

Svergun, D., Barberato, C., and Koch, M.H.J. (1995). CRYSOL - a program to evaluate X-ray solution scattering of biological macromolecules from atomic coordinates. J. Appl. Crystallogr. *28*, 768–773.

Tafel, A.A., Wu, L., and McHugh, P.J. (2011). Human HEL308 localizes to damaged replication forks and unwinds lagging strand structures. J. Biol. Chem. *286*, 15832–15840.

Thoma, N.H., Czyzewski, B.K., Alexeev, A.A., Mazin, A.V., Kowalczykowski, S.C., and Pavletich, N.P. (2005). Structure of the SWI2/SNF2 chromatin-remodeling domain of eukaryotic Rad54. Nat. Struct. Mol. Biol. *12*, 350–356.

Truong, L.N., Li, Y., Shi, L.Z., Hwang, P.Y., He, J., Wang, H., Razavian, N., Berns, M.W., and Wu, X. (2013). Microhomology-mediated end joining and homologous recombination share the initial end resection step to repair DNA double-strand breaks in mammalian cells. Proc. Natl. Acad. Sci. USA *110*, 7720–7725.

Velankar, S.S., Soultanas, P., Dillingham, M.S., Subramanya, H.S., and Wigley, D.B. (1999). Crystal structures of complexes of PcrA DNA helicase with a DNA substrate indicate an inchworm mechanism. Cell *97*, 75–84.

Yoon, J.H., Roy Choudhury, J., Park, J., Prakash, S., and Prakash, L. (2014). A role for DNA polymerase theta in promoting replication through oxidative DNA lesion, thymine glycol, in human cells. J. Biol. Chem. *289*, 13177–13185.

Yousefzadeh, M.J., and Wood, R.D. (2013). DNA polymerase POLQ and cellular defense against DNA damage. DNA Repair (Amst.) *12*, 1–9.

Yousefzadeh, M.J., Wyatt, D.W., Takata, K., Mu, Y., Hensley, S.C., Tomida, J., Bylund, G.O., Doublie, S., Johansson, E., Ramsden, D.A., et al. (2014). Mechanism of suppression of chromosomal instability by DNA polymerase POLQ. PLoS Genet. *10*, e1004654.

Zahn, K.E., Averill, A.M., Aller, P., Wood, R.D., and Doublie, S. (2015). Human DNA polymerase theta grasps the primer terminus to mediate DNA repair. Nat. Struct. Mol. Biol. *22*, 304–311.

Structure, Volume *23*

Supplemental Information

Structure of the Helicase Domain of DNA Polymerase Theta Reveals a Possible Role in the Microhomology-Mediated End-Joining Pathway

Joseph A. Newman, Christopher D.O. Cooper, Hazel Aitkenhead, and Opher Gileadi

Supplementary Material

Supplementary Figures

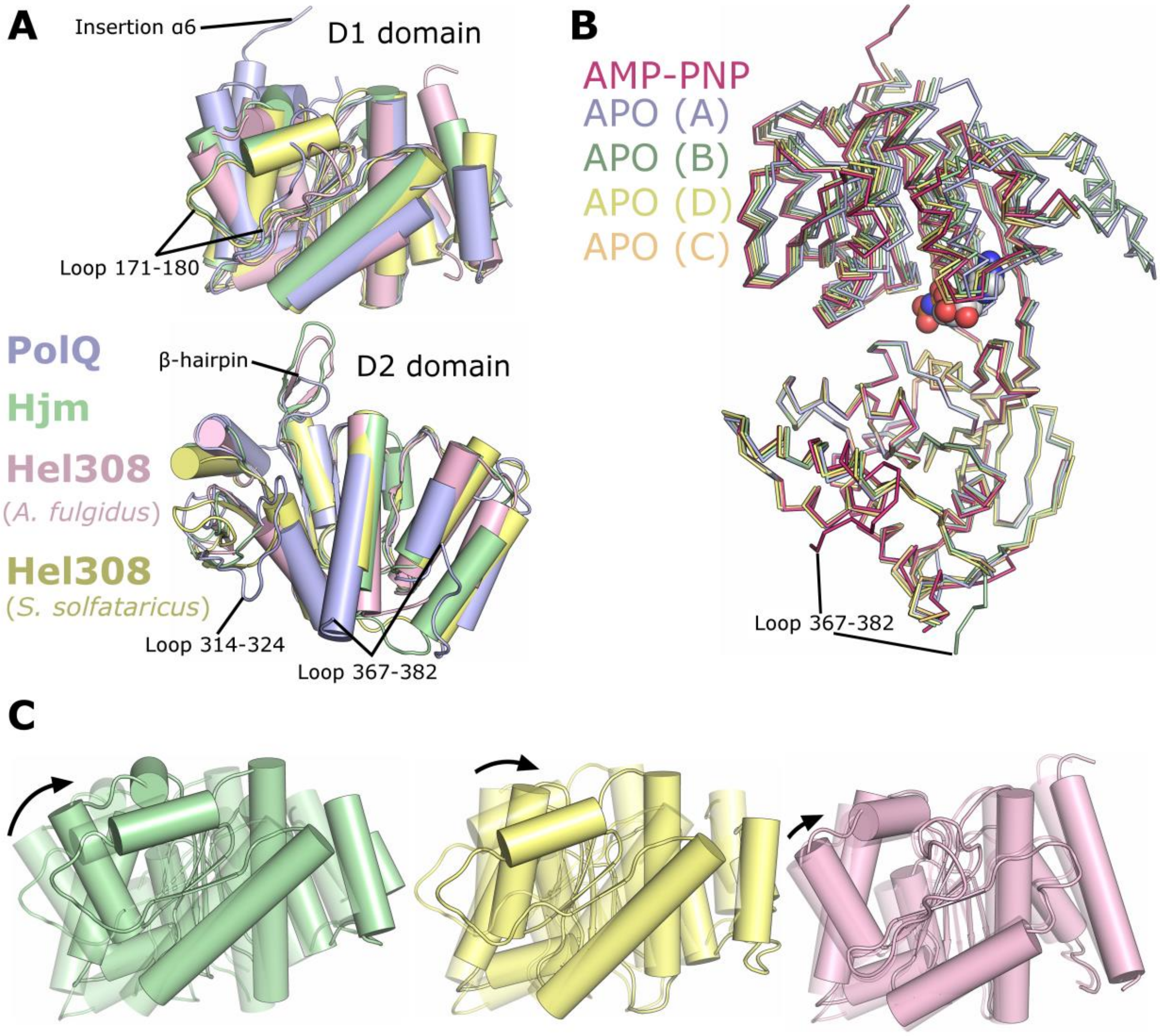

Figure S1, related to Figure 1- Comparison of Polθ-HLD core with archaeal Hel308/Hjm proteins. (**A**) Structural superposition of Polθ-HLD (blue), Hjm (green), *A. fulgidus* Hel308 (pink), and *S. solfataricus* Hel308 (yellow), on the basis of the D1 (upper) and D2 (lower) domains, with variable regions labelled. (**B**) Comparison of the D1-D2 conformation of the nucleotide and the 4 chains of the APO protein. All structures were superposed on the basis of the D2 domain alone. (**C**) Comparison of the D1-D2 domain conformations of Hjm (green), *S. solfataricus* Hel308 (yellow) and *A. fulgidus* Hel308 (pink) with PolQ. The semi-transparent image shows the position of the D1 domain when a structural superposition to PolQ is performed on the basis of the D2 domain alone, the solid image shows the altered position when superposed on the basis of the D1 domain alone. The black arrow shows an approximate vector describing the relative domain motion (shorter arrow is more similar conformation).

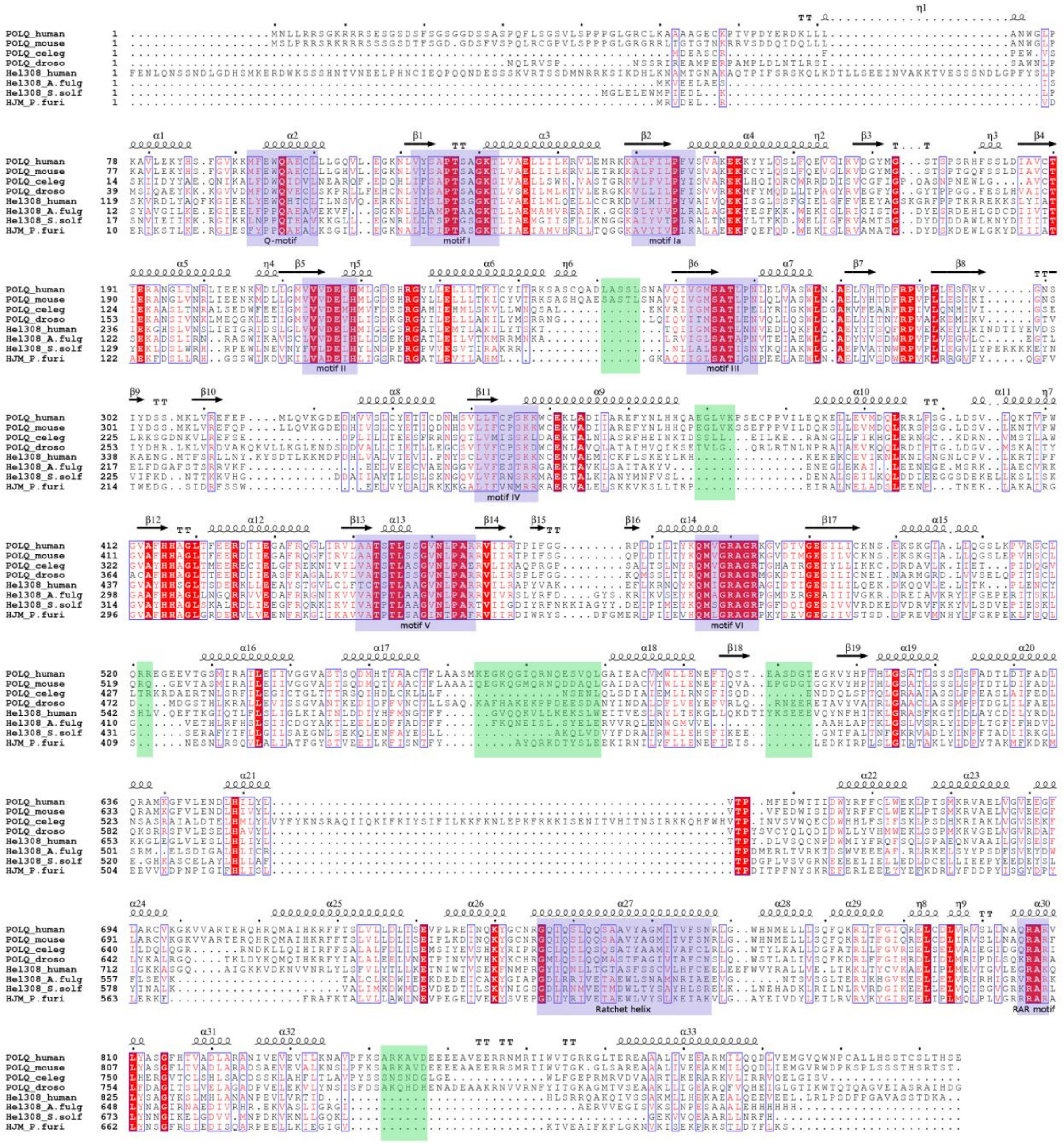

Figure S2, related to Figure 1B – Multiple sequence alignment of Polθ-HLD and selected homologues. Multiple sequence alignment was performed using CLUSTALW, and displayed using ESCRIPT. Secondary structural elements from the Polθ-HLD AMP-PNP structure (PDBid 5AGA) are displayed on top with important helicase regions highlighted in purple, and regions disordered in the 5AGA structure highlighted in green.

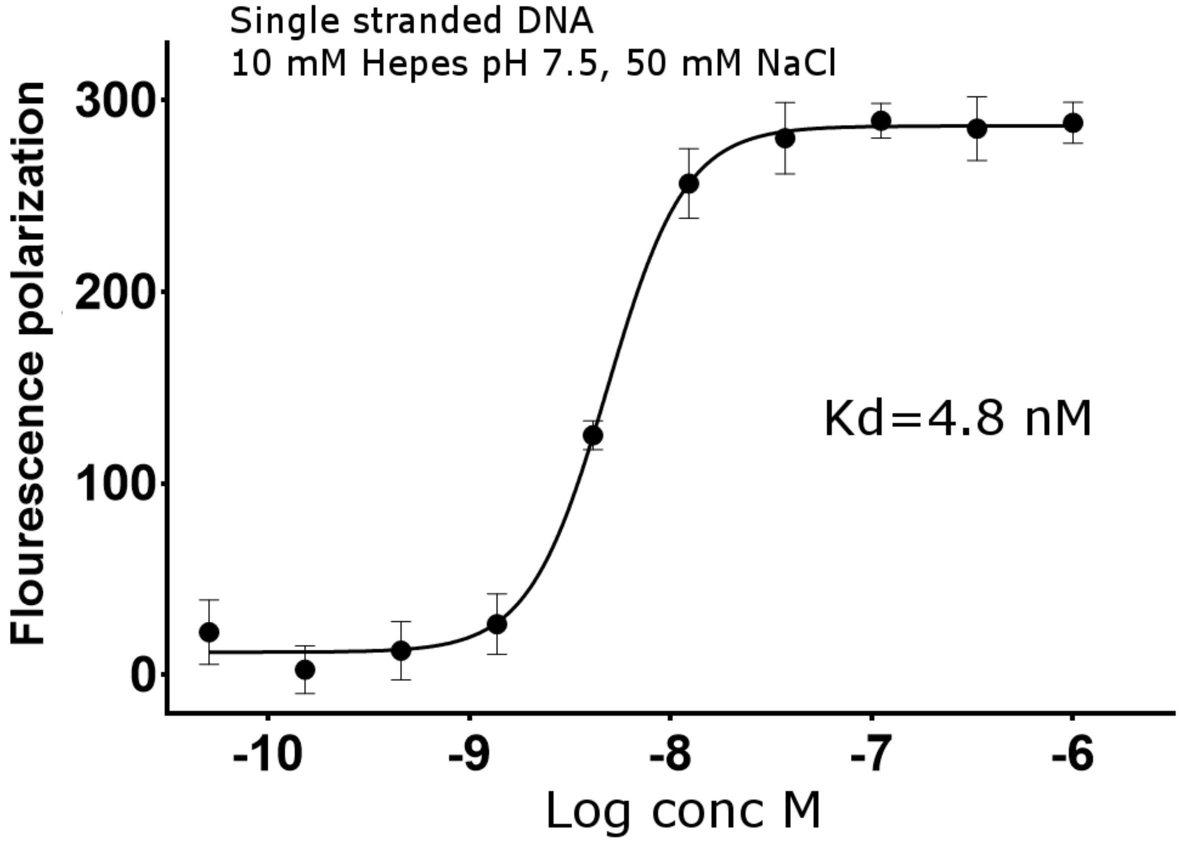

Figure S3, related to Figure 5- Polθ-HLD binding to single stranded DNA in low salt buffer. Error bars are plotted ± standard error of at least three independent replicates.

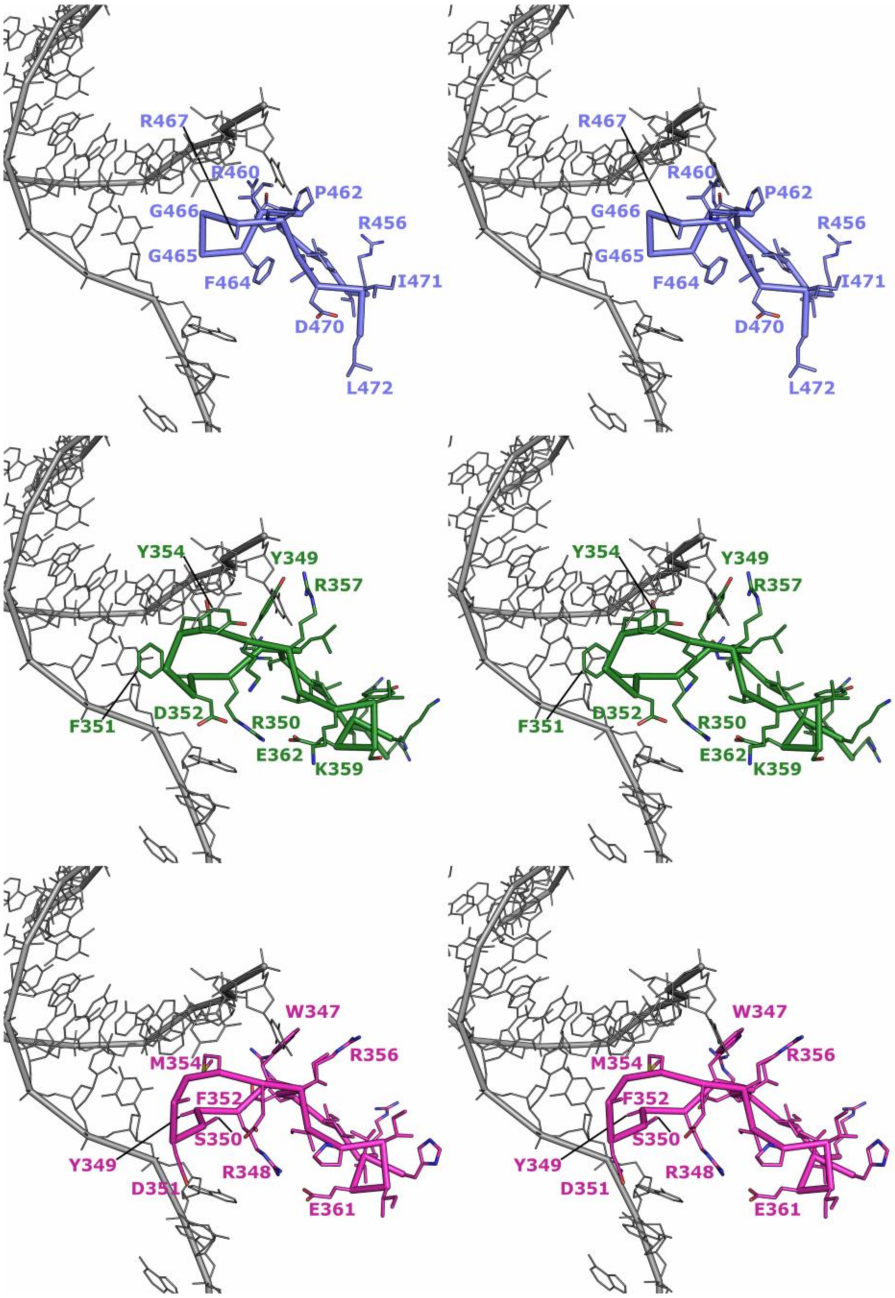

Figure S4, related to Figure 6- Comparison of the β-hairpins of Polθ-HLD, *A. fulgidus* Hel308 and Hjm. Stereo view of the β-hairpin regions of Polθ-HLD (blue, upper panel), *A. fulgidus* Hel308 (green, centre panel) and Hjm (pink, lower panel) with key residues labelled. The DNA from the *A. fulgidus* Hel308 DNA complex is shown in grey for reference throughout.

Table S1A, related to Experimental Procedures: DNA-binding assays. Sequences of oligonucleotides used in the fluorescence polarisation DNA binding assay

Oligonucleotide name	Sequence
POLQ-1	*5'ATCGATAGTCGGATCCTCTAGACAGCTCCATGTAGCAAGGCACTGGTAGAATTCGGCAGCGTC (63 bases)
POLQ-2	5'GACGCTGCCGAATTCTACCAGTGCCTTGCTACATGGAGCTGTCTAGAGGATCCGACTATCGAT (63 bases)
POLQ-3	5'GACGCTGCCGAATTCTACCAGTGCCTTGCTAGGACATCTTTGCCCACCTGCAGGTTCACCC (61 bases)
POLQ-4	5'GGAGCTGTCTAGAGGATCCGACTATCGA (28 bases)
POLQ-5	5'TGGGTGAACCTGCAGGTGGGCAAAGATG (28 bases)
POLQ-6	5'GGGTGAACCTGCAGGTGGGCAAAGATGTCCATTAGTGGATCCTTAGCACCGTTGTAAGACG (61 bases)
POLQ-7	5'CGTCTTACAACGGTGCTAAGGATCCACTAATCATGGAGCTGTCTAGAGGATCCGACTATCGAT (63 bases)
POLQ-8	5'GACGCTGCCGAATTCTACCAGTGCCTTGCTATTTTTTTTTTTTTCATGGAGCTGTCTAGAGGATCCGACTATCGAT (75 bases)
POLQ-9	5'GACGCTGCCGAATTCTACCAGTGCCTTG (28 bases)

* Oligonucleotide POLQ-1 was obtained with a Fluorescein isothiocyanate label on the 5' end

Table S1B, related to Experimental Procedures: DNA-binding assays. Mixing scheme for DNA substrates

Substrate name	Structure	Oligonucleotides
Single stranded	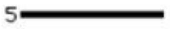	POLQ-1
Double stranded	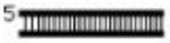	POLQ-1 + POLQ-2
3′ Overhang	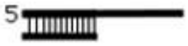	POLQ-1 + POLQ-4
Splayed duplex	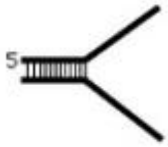	POLQ-1 + POLQ-3
3′ Flap	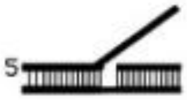	POLQ-1 + POLQ-3 + POLQ-4
3 way junction	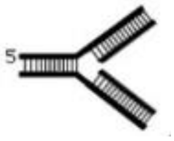	POLQ-1 + POLQ-3 + POLQ-4 + POLQ-5
4 Way junction	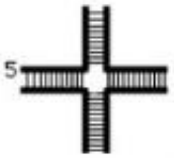	POLQ-1 + POLQ-3 + POLQ-6 + POLQ-7
Looped duplex	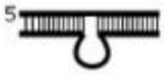	POLQ-1 + POLQ-8
5′ overhang	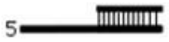	POLQ-1 + POLQ-9

In all cases the fluorophore was attached to the 5′ end of oligo POLQ-1 in the position marked by the 5 on the structure diagrams.

Supplemental Experimental Procedures

Protein expression and purification.

The Polθ-HLD constructs (residues 1-894 and 67-894) were cloned in to the baculovirus transfer vector pFB-LIC-Bse and transformed into *E. coli* DH10Bac to generate recombinant bacmids. Spodoptera frugiperda (Sf9) cells were infected with P2 virus (2x 10^6 cells/ml), and proteins were expressed in 1 litre cultures at 27°C for 72hrs and harvested by centrifugation. For purification cell pellets were thawed and resuspended in buffer A (50 mM HEPES pH 7.5, 500 mM NaCl, 5% glycerol, 10 mM imidazole, 0.5 mM Tris (2-carboxyethyl) phosphene (TCEP)), with the addition of 1x protease inhibitor set VII (Merck, Darmstadt, Germany). Cells were lysed using sonication and cell debris pelleted by centrifugation. Lysates were applied to a Ni-IDA IMAC gravity flow column, washed with 2 column volumes of wash buffer (buffer A supplemented with 30 mM imidazole), and eluted with the addition of 300 mM imidazole in buffer A. The purification tag was cleaved with the addition of 1:20 mass ratio of His-tagged TEV protease during overnight dialysis into buffer B (20 mM HEPES, pH 7.5, 500 mM NaCl, 5 % glycerol, 0.5 mM TCEP). TEV was removed by IMAC column rebinding and final protein purification was performed by size exclusion chromatography using a HiLoad 16/60 Superdex 200 column at 1 ml/min in buffer B. Protein concentrations were determined by measurement at 280nm (Nanodrop) using the calculated molecular mass and extinction coefficients.

Crystallization and Structure Determination

For crystallization Polθ-HLD was concentrated to 10 mg/ml using a 50,000 mwco centrifugal concentrator and exchanged to a buffer containing 10 mM HEPES pH 7.5, 250 mM NaCl, 0.5 mM TCEP. AMP-PNP and ADP bound crystals were obtained at 20°C from conditions containing 19% PEG 3350, 0.2M potassium citrate tribasic pH 8.5, 10 mM $MgCl_2$ and 10 mM of either AMP-PNP or ADP respectively. The *apo* protein was concentrated to 15 mg/ml and crystallized at 20°C from conditions containing 0.2 M NaCl, 0.1 M HEPES pH 7.0, 20 % PEG 6K and 10% ethylene glycol. Crystals were cryo-protected by transferring to a solution of mother liquor supplemented with 25 % ethelene glycol and flash-cooled in liquid nitrogen. Data were collected at diamond light source beamline I04-1 (AMP-PNP and ADP) and I03 (*apo*). Diffraction data were processed with the program XDS(Kabsch, 2010), and the structures were solved by molecular replacement using the program PHASER(McCoy et al., 2007) with *P.furiosis* Hjm(Oyama et al., 2009) structure as a starting model. Model building and real space refinement were performed in COOT(Emsley et al., 2010) and the structures refined using PHENIX REFINE(Adams et al., 2010). The structural coordinates and structure factors were

deposited in the Protein Data Bank, PDB: 5A9J (apo), 5AGA (AMP-PNP) and 5A9F (ADP). Structure validation summary reports can be retrieved from the PDB entries.

Analytical Size Exclusion Chromatography

Analytical size exclusion chromatography with on-line multiangle light scattering (SEC-MALS) was performed on a Viscotek TDA 305 system (Malvern Instruments Ltd) using a 15-ml Shodex KW-803 silica column (GE healthcare). 50 µl of the respective proteins were injected on to the column which was run at a flow rate of 0.5 ml/min in a buffer containing 20 mM HEPES 7.5, 5% glycerol, 500 mM NaCl and 0.5 mM TCEP. Calibration was performed using the molecular weight standards thyroglobulin (670,000 Da), ɣ-globulin (158,000 Da), ovalbumin (44,000 Da), myoglobin (17.000 Da) and vitamin B_{12} (1,350 Da), using the same buffer and flow rate. MALS data were analysed using the Omnisec software (Malvern Instruments Ltd) using Bovine serum albumin as an internal calibration sample.

Analytical Ultracentrifugation

Sedimentation velocity AUC experiments were performed on a XL-I Analytical Ultracentrifuge using a Ti-50 rotor and cells with double-sector centrepieces (Beckman Coulter, Brea, CA, USA). Experiments were performed with 0.35 mg/ml protein in 20 mM HEPES (pH 7.5), 150 mM NaCl, and 0.5 mM TCEP. Samples were filtered through a 0.22 µm syringe filter prior to analysis, employing a rotor speed of 40,000 rpm at 20°C, against a matched buffer reference. Absorbance (280 nm) and interference data were analyzed with SEDFIT(Schuck, 2000) to calculating c(s) distributions, and using partial specific volumes and buffer parameters calculated using the program SEDNTERP(Harding et al., 1992). Theoretical sedimentation coefficients of model proteins were calculated from Polθ-HLD structures using the program HYDROPRO(Ortega et al., 2011).

Small angle X-ray scattering

Small angle X-ray scattering measurements of Polθ-HLD in solution were performed at Diamond light source beamline B21 using a BIOSAXS robot for sample loading. Measurements were made using protein concentrations of 2.5, and 1.25 mg/ml in a buffer comprising 20 mM HEPES pH 7.5, 250 mM NaCl, 0.5 mM TCEP, 1 mM ADP. The data were reduced and buffer contributions subtracted with the DawnDiamond software suite and analysed using the program SCATTER (www.bioisis.net). Real space scattering profiles of atomic models were calculated from atomic models using CRYSOL(Svergun et al., 1995) and aligned and scaled to the experimental data using PRIMUS(Konarev et al., 2003). The agreement between theoretical and experimental scattering profiles was evaluated using the χ^2 free procedure(Rambo and Tainer, 2013) implemented in the program SCATTER.

DNA binding assays

DNA binding was measured using a fluorescence polarisation based assay. DNA oligonucleotide substrates were prepared by mixing the oligonucleotide sequences and combinations listed in table S1. For all substrates the master strand was labelled on the 5' end with Fluorescein isothiocyanate and oligonucleotides were mixed in the ratio 1:1.1 (slight excess on unlabelled oligonucleotide) at 5 mM final concentration in a buffer consisting of 10 mM HEPES pH 7.5, 50 mM NaCl before heating to 96°C and allowing to cool on a heat block over 2 hrs. Probes were used at a final concentration of 10 nM and binding experiments were performed in a buffer containing 10 mM HEPES pH 7.5, 150 mM NaCl. Measurements were performed in 96 well plates (200 µl volume) in a POLARstar omega plate reader (BMG Labtech). Kinetic constants were calculated from binding curves using a 4 parameter logarithmic binding equation using the program PRISM (GraphPad).

ATPase activity assays

ATPase activity of Polθ-HLD was measured using a pyruvate kinase, lactate dehydrogenase enzyme linked absorbance assay(Norby, 1988) in which the loss of absorbance at 340 nM (6,250 cm^{-1} M^{-1}) is coupled to ATP hydrolysis. The reaction mixtures (40 ul) contained 0.85 U Lactate Dehydrogenase, 4.2 U Pyruvate Kinase, 0.2 mM NADH, 0.5 mM Phosphoenolpyruvate, 20 mM HEPES pH 7.5, 200 mM NaCl, and ATP concentrations between 3 µM and 0.8 mM. For DNA stimulated ATPase the reaction mix contained in addition 2 µM of single stranded 18 bp DNA (ACTCGTCTCTAGCTTTTT). Reactions were initiated by the addition of protein (0.1 µM with DNA and 3.4 µM without) and measured over 15 minutes in a POLARstar omega plate reader (BMG Labtech). Kinetic constants were calculated from binding curves using a 4 parameter logarithmic binding equation using the program PRISM (GraphPad).

References

Adams, P.D., Afonine, P.V., Bunkoczi, G., Chen, V.B., Davis, I.W., Echols, N., Headd, J.J., Hung, L.W., Kapral, G.J., Grosse-Kunstleve, R.W., *et al.* (2010). PHENIX: a comprehensive Python-based system for macromolecular structure solution. Acta Crystallogr D Biol Crystallogr *66*, 213-221.

Emsley, P., Lohkamp, B., Scott, W.G., and Cowtan, K. (2010). Features and development of Coot. Acta crystallographica Section D, Biological crystallography *66*, 486-501.

Harding, S.E., Rowe, A.J., and Horton, J.C. (1992). Analytical ultracentrifugation in biochemistry and polymer science ([Cambridge]: Royal society of chemistry).

Kabsch, W. (2010). Xds. Acta crystallographica Section D, Biological crystallography *66*, 125-132.

Konarev, P.V., Volkov, V.V., Sokolova, A.V., Koch, M.H.J., and Svergun, D.I. (2003). PRIMUS: a Windows PC-based system for small-angle scattering data analysis. Journal of applied crystallography *36*, 1277-1282.

McCoy, A.J., Grosse-Kunstleve, R.W., Adams, P.D., Winn, M.D., Storoni, L.C., and Read, R.J. (2007). Phaser crystallographic software. Journal of applied crystallography *40*, 658-674.

Norby, J.G. (1988). Coupled Assay of Na+,K+-Atpase Activity. Method Enzymol *156*, 116-119.

Ortega, A., Amoros, D., and Garcia de la Torre, J. (2011). Prediction of hydrodynamic and other solution properties of rigid proteins from atomic- and residue-level models. Biophysical journal *101*, 892-898.
Oyama, T., Oka, H., Mayanagi, K., Shirai, T., Matoba, K., Fujikane, R., Ishino, Y., and Morikawa, K. (2009). Atomic structures and functional implications of the archaeal RecQ-like helicase Hjm. BMC structural biology *9*, 2.
Rambo, R.P., and Tainer, J.A. (2013). Accurate assessment of mass, models and resolution by small-angle scattering. Nature *496*, 477-481.
Schuck, P. (2000). Size-distribution analysis of macromolecules by sedimentation velocity ultracentrifugation and lamm equation modeling. Biophysical journal *78*, 1606-1619.
Svergun, D., Barberato, C., and Koch, M.H.J. (1995). CRYSOL - A program to evaluate x-ray solution scattering of biological macromolecules from atomic coordinates. Journal of applied crystallography *28*, 768-773.
